# Cerebrospinal delivery of a bidirectional AAV9 vector improves optic nerve and retinal pathology in a sheep model of Tay-Sachs disease

**DOI:** 10.1016/j.ymthe.2026.06.018

**Published:** 2026-06-11

**Authors:** Brett D. Story, Sara M. Thomasy, Sara A. Adelman, Toloo Taghian, Yvette Lopez, Monica Ardon, Hikaru Shiraishi, Sabina Khan, Meher Khan, Maria Do, Hector R. Benatti, Stephanie Bertrand, Rachel Prestigiacomo, Rachael Gately, Douglas R. Martin, Miguel Sena-Esteves, Jun Xie, Guangping Gao, Leandro B.C. Teixeira, Gillian C. Shaw, Nicholas Marsh-Armstrong, Heather L. Gray-Edwards

**Affiliations:** 1 Department of Surgical and Radiological Sciences, School of Veterinary Medicine, University of California–Davis, Davis, CA 95616, USA; 2 Department of Ophthalmology & Vision Science, University of California, Davis, Sacramento, CA 95817, USA; 3 Department of Genetic and Cellular Medicine, University of Massachusetts Medical School, Worcester, MA 01605, USA; 4 Tufts Farm, Cummings School of Veterinary Medicine, Tufts University, North Grafton, MA 01536, USA; 5 Department of Pathobiological Sciences, School of Veterinary Medicine, University of Wisconsin-Madison, Madison, WI 53706, USA; 6 Department of Ambulatory and Theriogenology, Cummings School of Veterinary Medicine, Tufts University, North Grafton, MA 01536, USA; 7 Scott-Ritchey Research Center, Auburn University College of Veterinary Medicine, Auburn, AL 36849, USA; 8 Department of Radiology, University of Massachusetts Medical School, Worcester, MA 01605, USA

## Abstract

Tay-Sachs disease (TSD) is a fatal neurodegenerative lysosomal storage disease. The Jacob sheep is the only large-animal model of TSD, yet ocular pathology and the therapeutic potential of gene therapy remain poorly defined. Sheep cohorts included normal controls (*n* = 3); untreated TSD-affected (*n* = 4); intravenous AAV9-Bic_HexA/HexB-treated (*n* = 3); and intracerebroventricular, cisterna magna, and lumbar intrathecal AAV9-Bic_HexA/HexB-treated sheep (cerebrospinal fluid [CSF] therapy; *n* = 7). Retinal histopathology and immunohistochemistry, retinal whole-mount analyses for retinal ganglion cell (RGC) morphology and density, optic nerve evaluation with *p*-phenylenediamine (PPD )semi-thin sections, qPCR assessment for vector genomes, and RNAscope probes for transgene expression were performed. Untreated TSD sheep exhibited RGCs with abundant microvesicular cytoplasmic expansion and optic nerve spheroids, with storage material variably staining with periodic acid-Schiff. Marked astrocytosis, microgliosis, and GM2 accumulation within RGCs were present. Optic nerve axon counts and RGC density were significantly reduced, and optic nerve damage scores increased, in untreated and IV-treated sheep but were rescued with short-term CSF therapy. GM2 volume and signal intensity per RGC were significantly reduced following short-term CSF therapy. Minimal but detectable retinal vector genomes and transgene expression were observed. These findings demonstrate retinal and optic nerve pathology in Jacob sheep with TSD and AAV9 therapy.

## INTRODUCTION

The GM2 gangliosidoses (Tay-Sachs disease [TSD] and Sandhoff disease) are rare, autosomal recessive, fatal, neurodegenerative lysosomal storage disorders caused by a deficiency of the enzyme β-N-acetylhexosaminidase (Hex). Hex exists as a heterodimer encoded by *HEXA* (α subunit) and *HEXB* (β subunit), and mutations in these genes result in TSD and Sandhoff disease, respectively. TSD has been classified based on age presentation into infantile, juvenile, and late-onset forms. The large majority of TSD cases are classified as infantile, with the juvenile and late-onset forms being rarer.^1^ In contrast to the infantile form, which usually leads to death between 2 and 5 years of life due to severe progressive neurological disease, juvenile and late-onset subtypes have a more protracted, yet relentless, clinical course.^2^ Jacob sheep are the only animal model that share similar genetics, biochemical pathology, and disease progression with TSD patients, and they have a central nervous system (CNS) similar in size and complexity to that of children.^3-7^

A hallmark fundoscopic clinical finding in human patients, particularly in infantile forms of TSD, is the appearance of a cherry-red spot in the central fovea. This finding is inconsistently observed across juvenile subtypes, with vision loss occurring much later than in infantile forms. Instead, retinitis pigmentosa and optic nerve atrophy can be seen late in the disease course in juvenile patients.^8^ Cherry-red spots have been noted as early as 2 days of life and are present in almost all infantile patients by 6 months of age. Vision reduction occurs by 12–18 months of age, and, by 30 months, most infantile patients become blind.^9^ In one study, 90% of infantile patients with GM2 gangliosidosis (TSD, Sandhoff disease) exhibited cherry-red spots.^10^

Novel treatment options for TSD are on the horizon, with development and optimization occurring in animal models.^11^ Over the last few decades, substantial advances have been made in harnessing adeno-associated virus (AAV) vector-mediated gene delivery to treat ocular disorders.^12-14^ The efficacy of an AAV rhesus isolate 8 (AAVrh8)-based gene therapy in the Jacob sheep model for TSD and a feline Sandhoff model has been studied.^15-19^ Therapeutic efficacy in these large-animal models has led to the first tests of a dual-vector AAV gene therapy for TSD and Sandhoff disease in humans (IND#16225; clinical trials.gov NCT04669535). Early clinical results are promising; however, this approach requires bilateral thalamic injections and is limited to infantile patients.^20,21^ We have developed a bidirectional vector that encodes both genes and can be used by cerebrospinal fluid (CSF) or intravenous injection. Preliminary studies show profound efficacy in rodent and sheep models.^22,23^ As such, an opportunity exists in this large-animal model to fully evaluate the ocular manifestations of this neurodegenerative disease and the effects of this gene-therapy approach.

Apart from limited evaluations in physician^24-27^ and veterinary medicine,^28-32^ many facets of ocular pathology in gangliosidosis remain unknown. The Jacob sheep provides an invaluable animal model for understanding the overall pathology of naturally occurring TSD in this species. A missense mutation in the *HEXA* cDNA causes TSD in the Jacob sheep,^4^ and a thorough evaluation of the ocular pathology in sheep will significantly further our understanding of the pathophysiology associated with this disease. Future therapies are imminent in the coming years for human patients; therefore, an accurate knowledge of ocular lesions and potential improvement with AAV9-Bic_HexA/HexB therapy is pertinent.

The purpose of this study is to determine retinal and optic nerve lesions in a Jacob sheep model of TSD and to establish whether gene therapy improves neuroinflammation and retinal changes. We sought to investigate whether Jacob sheep with naturally occurring TSD exhibit retinal lesions similar to those observed in humans and other animal models of gangliosidosis. We expected marked changes to the inner retina and optic nerve. We additionally hypothesized that there would be minimal changes in retinal and optic nerve histopathology in AAV-treated (via intravenous or CSF therapy—cisterna magna, intracerebroventricular, lumbar intrathecal) TSD sheep versus untreated TSD sheep.

## RESULTS

### Gross ocular anatomy is normal in untreated and treated TSD sheep

Grossly, all eyes were normal with no discernible ocular defects. During retinal dissection, the anterior segment and retina were grossly examined for each eye. There were no gross pathological findings of the cornea, ciliary body, lens, or retina (Figures S1 and S2) in any sheep. Furthermore, two affected TSD sheep (a 7-month endpoint male and a 9-month endpoint female) had entire globes processed for ocular histopathology (Figure S1) with no evidence of anterior segment pathology.

### Histopathology shows normal retinal thickness with intracytoplasmic inclusions in retinal ganglion cells and optic nerve spheroid formation

The total retinal thickness was analyzed in the superotemporal peripheral and superotemporal midcentral retina, and no significant difference was observed between groups. However, when evaluating individual retinal layers across all groups, a significantly decreased thickness was appreciated in the inner nuclear layer (INL), specifically in untreated controls and IV-treated sheep (*p* = 0.04) when compared to normal sheep. Additionally, when evaluating the midcentral RGCL thickness, a mild but significant increase in thickness was seen in TSD sheep compared to untreated controls and short-term CSF sheep (*p* = 0.04). Short-term CSF sheep also showed mild but significant decreases in inner plexiform layer (IPL) (*p* = 0.04) and outer plexiform layer (OPL) (*p* = 0.02) thickness compared with controls. There were no significant differences when comparing other individual retinal layers (*p* > 0.05) (Figures S3 and S4).

With histopathology, retinal ganglion cells (RGCs) demonstrated diffuse, abundant amphophilic to eosinophilic cytoplasm expanded by profuse microvesicles; the optic nerve contained scattered spheroids in untreated TSD sheep (Figure S5). The cytoplasm of RGCs within the INL stain variably pink with periodic acid-Schiff (PAS) stain in untreated TSD sheep; spheroids in the optic nerve also stained variably pink with PAS stain (Figure S6). By contrast, the RGC cytoplasm in normal sheep retinas was clear or faint pink with PAS stain. In the majority of untreated TSD sheep, large cells in the INL were present with abundant microvacuolated cytoplasm, which may represent displaced RGCs or possibly amacrine cells. The remaining retinal layers were within normal limits including the photoreceptors. The retinal pigmented epithelium (RPE) of all sheep showed vacuolization (Figure S7). Together, these data demonstrate RGC pathology including optic nerve axonal degeneration in TSD sheep.

### Neuroinflammation was evident by inner retinal astrocytosis and microgliosis in TSD and AAV9-Bic_HexA/HexB-treated TSD sheep

**Figure 1.**
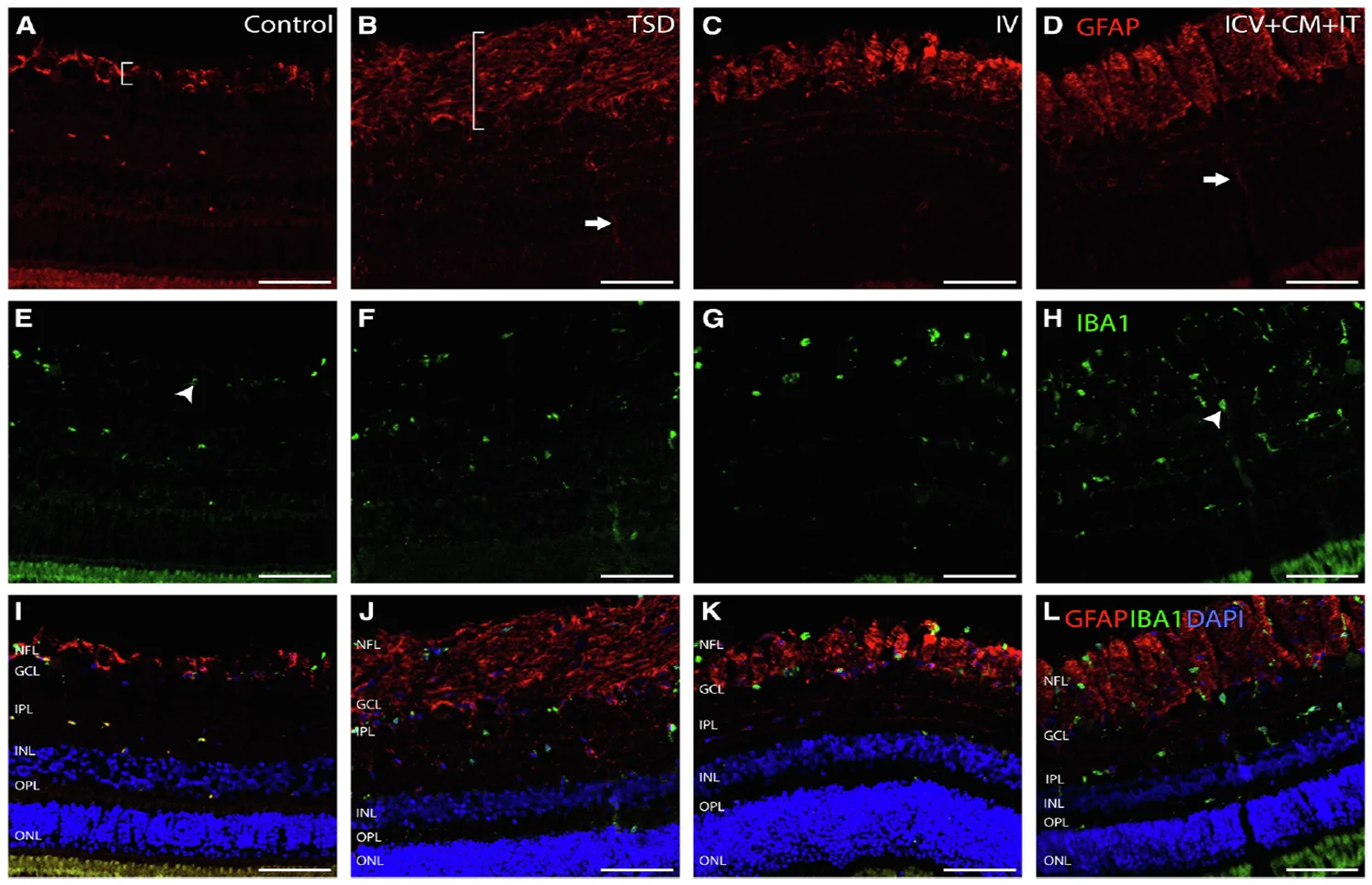
Astrocytosis and microgliosis in treated and untreated TSD sheep retinas In normal sheep, there is minimal retinal astrocytosis (bracket, A) and microgliosis (arrowhead, E) as indicated by GFAP and IBA1 staining patterns, respectively (A, E, and I). However, in TSD (B, F, and J), IV-treated (C, G, and K), and short-term CSF therapy sheep (D, H, and L), there is marked inner retinal astrocytosis (bracket, B, arrowheads B and D) and microgliosis (arrowhead, H). Reactive astrocytosis (TSD, IV, ICV + CM + IT) is characterized by increased GFAP immunoreactivity; hypertrophic, vertically oriented processes; and rentinal nerve fiber layer (RNFL) expansion. Increased IBA1-positive cells (TSD, IV, ICVM + CM + IT) with transition from ramified to amoeboid morphology is consistent with microglial activation. AAV9 therapy does not appear to ameliorate the retinal neuroinflammatory cell populations. GFAP, red; IBA1, green; DAPI, blue. Scale bar, 50 μm.

We assessed the retinal neuroinflammatory response by evaluating immunolabeled microglia (Iba1) and astrocytes (GFAP) using midcentral retinal sections (Figure 1). In normal sheep, resident astrocytes are seen in the nerve-fiber layer (NFL) with microglia signal observed throughout the inner retina. By contrast, abundant microglia and astrocytes are seen in both untreated and treated TSD sheep. The GFAP immunolabeling was increased in untreated and treated TSD sheep in the NFL and ganglion cell layer (GCL). Elongated Muller cells are seen traversing the NFL and GCL in TSD, IV-treated, and CSF-treated sheep. Likewise, Iba1 immunolabeling was increased in untreated and treated TSD sheep in the NFL, GCL, and IPL. In normal controls, microglial cells are ramified, indicative of being in a resting or homeostatic state. By contrast, microglial cells have an activated amoeboid (rounded) phenotype in treated and untreated TSD sheep. In aggregate, retinal neuroinflammation is marked in untreated and AAV9-Bic_HexA/HexB-treated TSD sheep; intravenous or CSF treatments do not appear to lessen the retinal inflammatory burden. These data illustrate that retinal neuroinflammation is marked in TSD and persistent in treated TSD sheep. Imaging data from the long-term CSF sheep cohort are available for review in Figure S8.

### TSD leads to optic nerve degeneration and axonal loss that is rescued with CSF therapy

**Figure 2.**
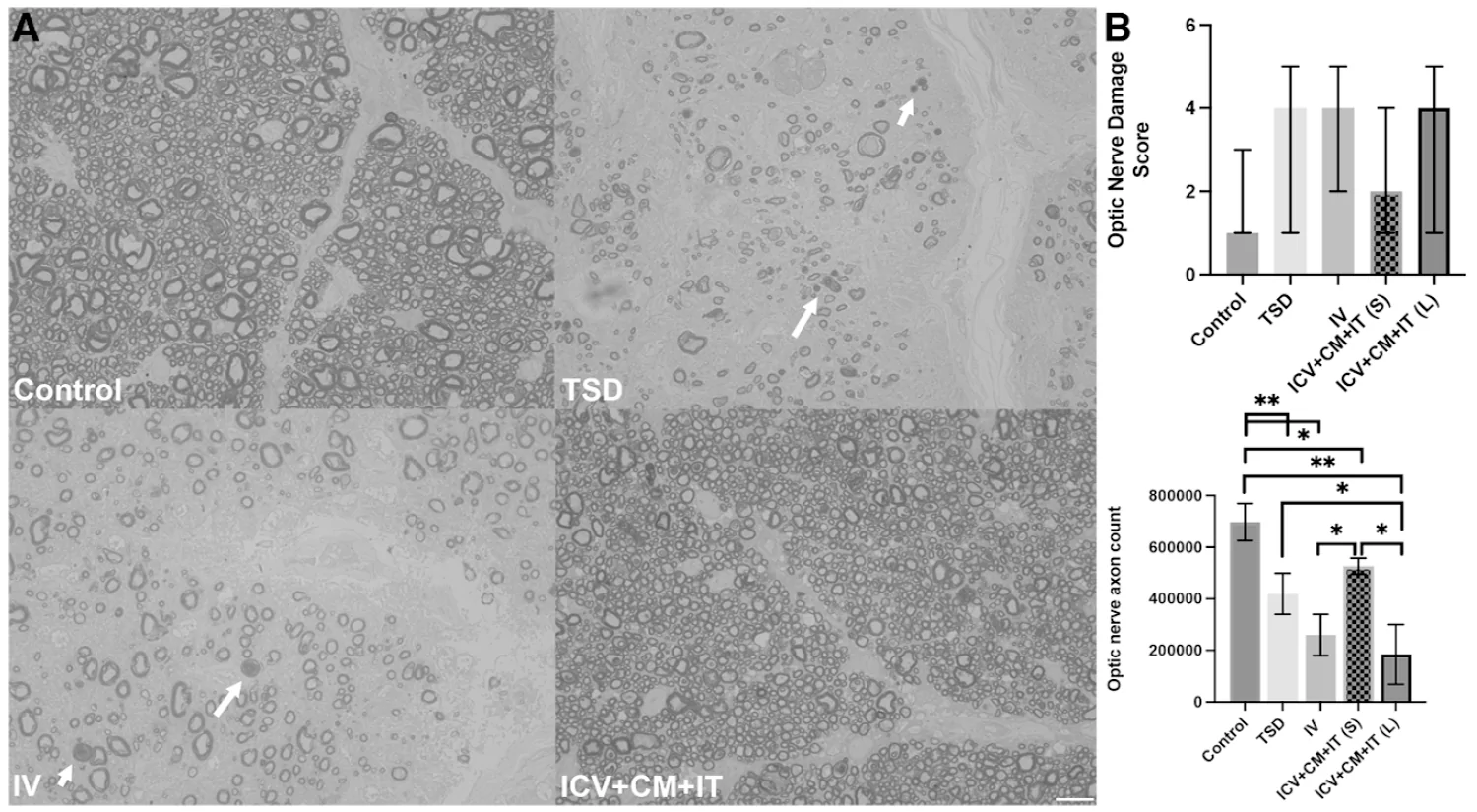
Optic nerves of ICV + CM + IT short-term-treated sheep show less axonal degeneration (A) Control sheep display normal axonal arrangement with few dark-staining axons and no identifiable axonal swelling or gliosis. By contrast, the axons in TSD and IV-treated sheep show a high percentage of axonal death with gliosis, darkly stained axons (white arrows), and occasional axonal swelling. Short-term CSF-therapy-treated sheep display an intermediate rescued phenotype. (B) Optic nerve damage is increased in TSD and IV treated sheep compared to controls. There is no difference in optic nerve damage between TSD and IV-treated sheep. Short-term CSF therapy sheep show improved damage scores compared to TSD and IV-treated sheep; however, this benefit is lost long term (Figure S8). Axon counts (per mm^2^) are significantly lower in TSD and IV-treated sheep compared to controls (*p = 0.007, **p = 0.002, respectively). Axon counts significantly increased in short-term CSF therapy sheep compared to IV treated (*p = 0.01) and approached significance in CSF-treated sheep and TSD (*p = 0.08). Long-term-therapy sheep have significantly fewer axons compared to normal controls (**p = 0.005), short-term CSF therapy sheep (*p = 0.03), and TSD sheep (*p = 0.04). Scale bar, 10 μm.

Since lysosomal storage disorders can lead to optic nerve damage, we examined the extent of optic nerve lesions and whether AAV9-Bic_HexA/HexB therapy improved neurodegeneration. Optic nerves were stained with *p*-phenylenediamine (PPD), to facilitate manual axon counting and assign optic nerve damage scores as PPD stains the myelin sheaths of healthy axons lightly while axoplasms of dying or damaged axons stain more darkly. We estimated that the normal optic nerve axon count in crossbred and Jacob sheep is 697,840 ± 72,125 axons, consistent with a previous report.^33^ Untreated TSD sheep, IV-treated sheep, and short- and long-term CSF-therapy TSD sheep had significantly reduced optic nerve axon counts (per mm^2^) of 419,092 ± 79,758 (*p* = 0.006), 259,693 ± 80,1890 (*p* = 0.002), 526,586 ± 31,260 (*p* = 0.04), and 184,730 ± 115,644 (*p* = 0.005), respectively. However, short-term CSF-therapy-treated TSD sheep had significantly greater axon counts when compared to IV-treated and long-term CSF-treated TSD sheep (*p* = 0.01 and *p* = 0.03; Figure 2B).

Histologically, PPD-stained optic nerve sections in TSD, IV-treated, and long-term CSF-treated TSD sheep demonstrated marked optic nerve degeneration along with gliosis. The axonal diameter was less uniform in size with the presence of many smaller-caliber axons compared to normal and short-term CSF-therapy-treated TSD sheep (Figure 2A). When we graded optic nerve damage scores, untreated TSD, IV-treated, and long-term CSF-treated TSD sheep had median optic nerve damage scores of 4 (range 1–5 for TSD and long-term CSF treated, 2–5 for IV; grading scale 1–5), indicating severe optic nerve degeneration. Consistent with axon counts, short-term CSF therapy in TSD sheep (median damage score 2, range 1–4) rescued optic nerve pathology (Figure 2B). Furthermore, Luxol fast blue (LFB) staining for myelin on optic nerve sections showed increased myelination in the short-term CSF therapy-treated TSD sheep (Figure S9). These data indicate that short-term CSF therapy-treated sheep show improved optic nerve axon counts with reduced degeneration.

### RGC counts are decreased in TSD and IV-treated sheep with significant GM2 storage and rescued by CSF therapy

**Figure 3.**
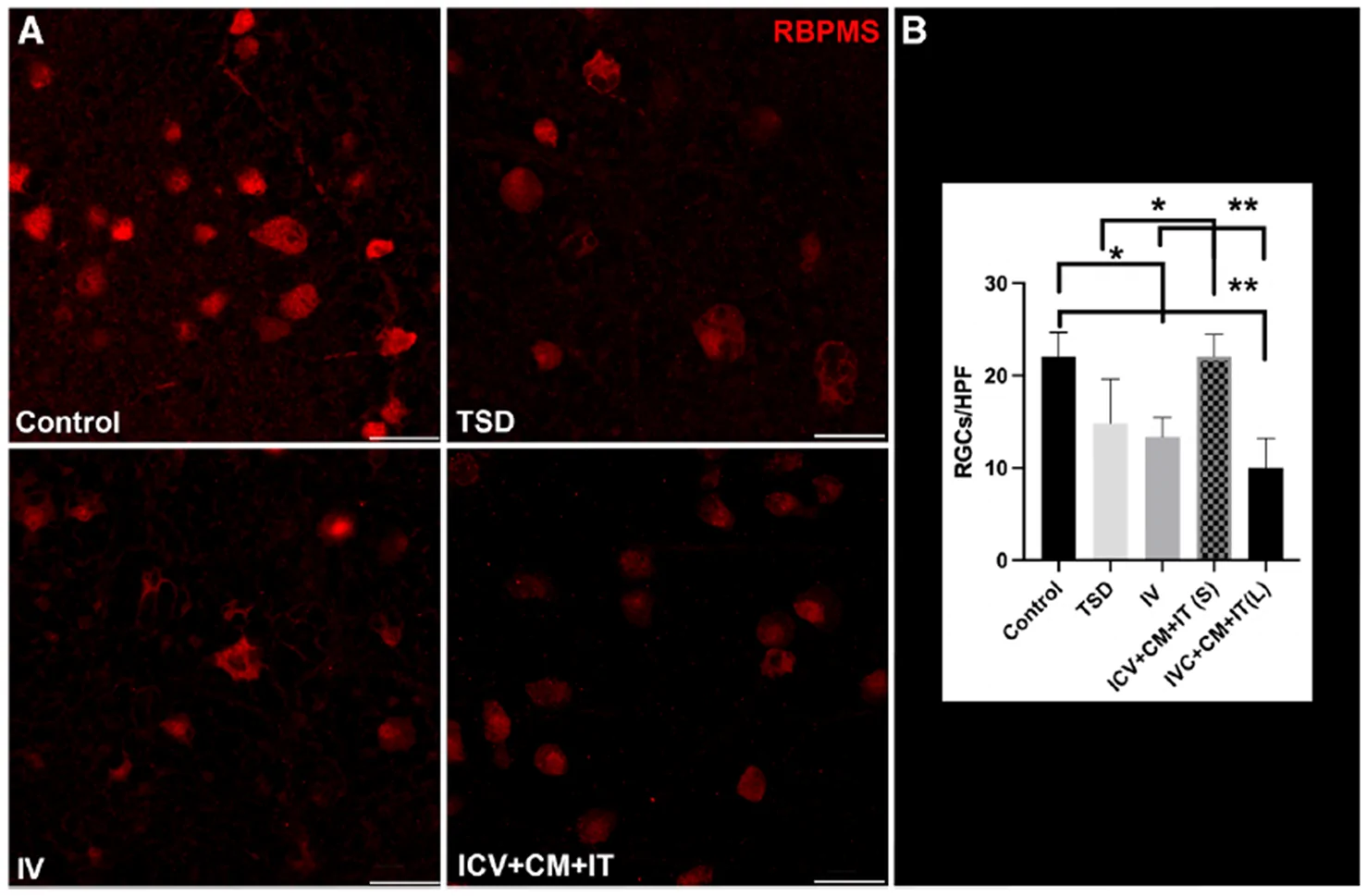
RGC soma density in the peripheral retina is rescued following short-term ICV + CM + IT CSF AAV9-Bic_HexA/HexB gene therapy (A and B) RGC density was significantly decreased based on RBPMS immunolabeling in IV and long-term CSF-treated sheep compared to controls (*p = 0.02; **p = 0.001), whereas RGC density was approaching significance in TSD sheep compared to controls (*p = 0.07). RGC density was normal in short-term CSF therapy compared to controls (*p = 0.81). Short-term CSF-therapy-treated sheep had significantly increased RGC density compared to TSD (*p = 0.04), IV-treated sheep (*p = 0.01), and long-term-CSF sheep (**p = 0.001). Scale bar, 50 μm. Red, RBPMS.

Retinal whole mounts immunolabeled for RNA-binding protein with multiple splicing (RBPMS) were analyzed to evaluate differences in RGC density per high power field (HPF) (Figure 3). Density of RGCs was significantly decreased in IV-treated sheep (13.3 ± 1.7 RGCs/HPF) and long-term CSF-treated sheep (10 ± 2.7 RGCs/HPF) compared to controls (22 ± 2.2 RGCs/HPF; *p* = 0.02 and *p* = 0.001, respectively) and approached significance in untreated TSD (14.8 ± 4.2 RGCs/HPF) sheep versus controls (*p* = 0.05). RGC density was rescued and normalized in short-term CSF therapy (22 ± 2.2 RGCs/HPF) when compared to untreated control RGC counts (*p* = 0.9). RGC density approached significance in short-term CSF-therapy-treated versus untreated sheep (*p* = 0.05) and was significantly increased compared to IV-treated sheep (*p* = 0.004). Lastly, sheep receiving short-term CSF therapy had significantly increased RGC densities compared with those receiving long-term CSF therapy (*p* = 0.001). In aggregate, short-term CSF therapy led to normal RGC density, while untreated TSD sheep, as well as those receiving IV or long-term CSF treatment with AAV9 gene therapy, had markedly reduced RGC densities.

**Figure 4.**
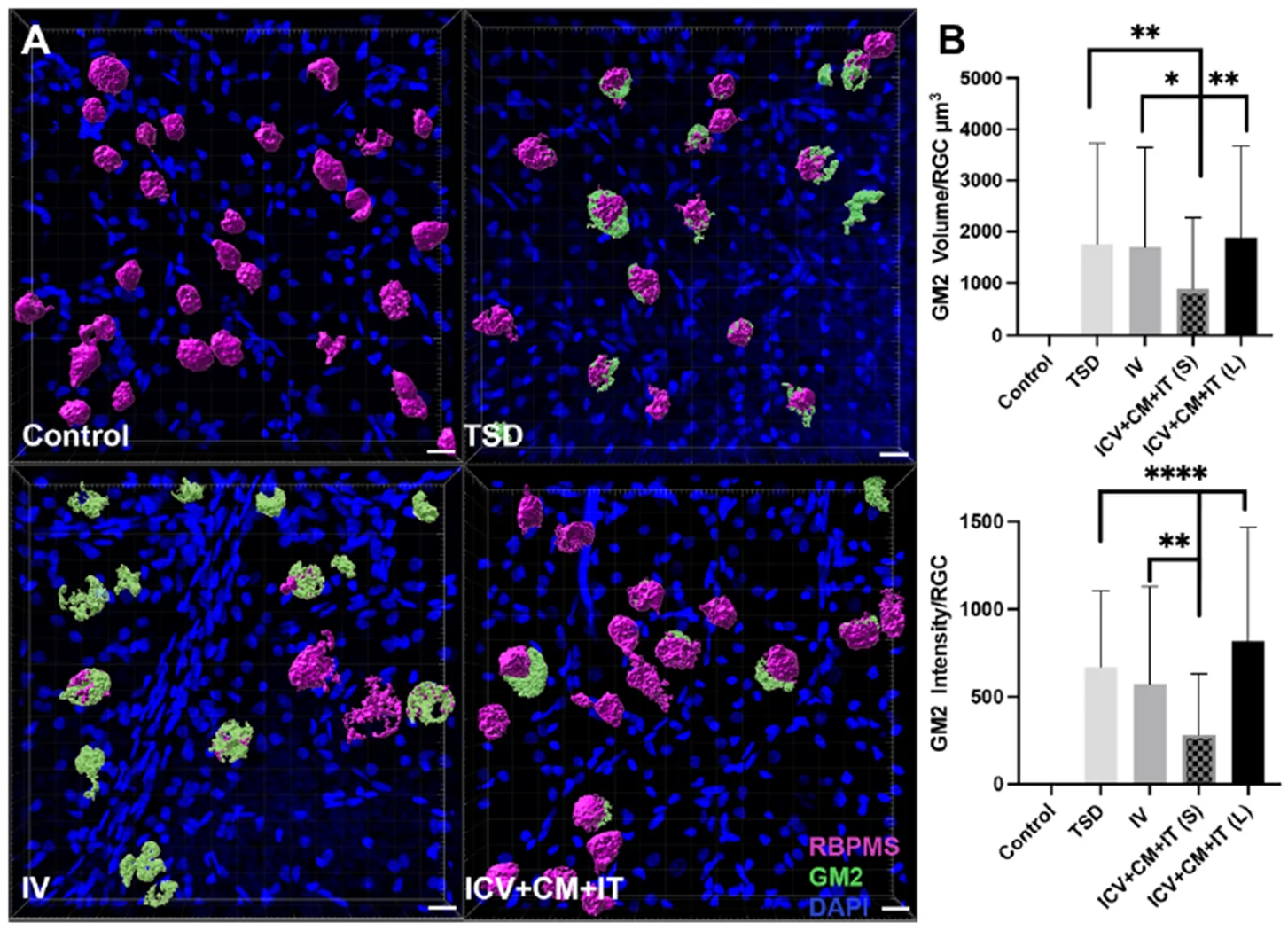
Short-term CSF-therapy-treated TSD sheep show significantly decreased GM2 in RGCs (A) Control sheep show no GM2 accumulation, whereas TSD and IV-treated sheep show GM2 accumulation in RBPMS-positive RGCs. Short-term CSF-treated sheep exhibit less GM2 accumulation. There was a statistically significant increase in GM2-positive, RBPMS-negative cells in TSD and IV-treated sheep. (B) GM2 volume per RBPMS-positive RGC is significantly reduced in short-term CSF therapy compared to TSD and IV-treated sheep (*p = 0.04, **p = 0.004). Similarly, GM2 intensity per RBPMS-positive RGC is significantly less in short-term CSF therapy compared to TSD and IV-treated sheep (**p = 0.002, ****p = 0.0001). RBPMS, magenta; GM2, green; DAPI, blue. Controls were significantly decreased compared to all groups (*p* < 0.0001). Compared to short-term CSF-treated sheep, long-term CSF-treated sheep had significantly increased GM2 volume and signal intensity (**p = 0.003, ****p = 0.0001). Scale bar, 20 μm.

Retinal whole mounts were then immunolabeled for RBPMS for RGC identification and anti-GM2 ganglioside to determine GM2 storage volume and distribution in affected RGCs. Normal sheep show no GM2 accumulation in RGCs or other retinal locations, while affected and treated sheep show varying degrees of GM2 accumulation in RGCs. The volume of GM2 in RBPMS-positive RGCs was significantly reduced (~50%) in short-term CSF therapy sheep (880.6 ± 1,376.8 μm^3^) compared to TSD (1,754.8 ± 1,863.6 μm^3^; *p* = 0.004), IV-treated (1,698.4 ± 1,922 μm^3^; *p* = 0.04), and long-term CSF-treated (1,886.5 ± 1,749.9; *p* = 0.003) sheep. Similarly, the signal intensity of GM2 in RBPMS-positive RGCs was significantly less in short-term CSF therapy sheep (282.6 ± 346.3) compared to TSD (668.9 ± 434.5; *p* = 0.0001) IV-treated (574.3 ± 548.1; *p* = 0.002), and long-term CSF-treated (818.3 ± 639.4; *p* = 0.0001) sheep (Figure 4).

Lastly, we noticed a propensity for GM2-positive, RBPMS-negative cells in TSD and IV-treated sheep retinas. Normal sheep (0 cells/HPF) showed significantly fewer GM2-positive RBPMS-negative cells compared to untreated TSD sheep (5 cells/HPF; *p* = 0.01). Short-term CSF therapy-treated sheep (1 cell/HPF) showed a significant decrease in the amount of GM2-positive, RBPMS-negative cells compared to untreated TSD sheep (5 cells/HPF; *p* = 0.04). All other groups showed no significant differences. In aggregate, these data indicate that TSD leads to significant GM2 accumulation in RGCs; short-term CSF therapy preserved RGC density and decreased GM2 volume distribution; and there was a higher propensity for GM2-positive, RBPMS-negative cells in TSD retinas.

### AAV9 vector distribution and HexA enzyme activity

**Figure 5.**
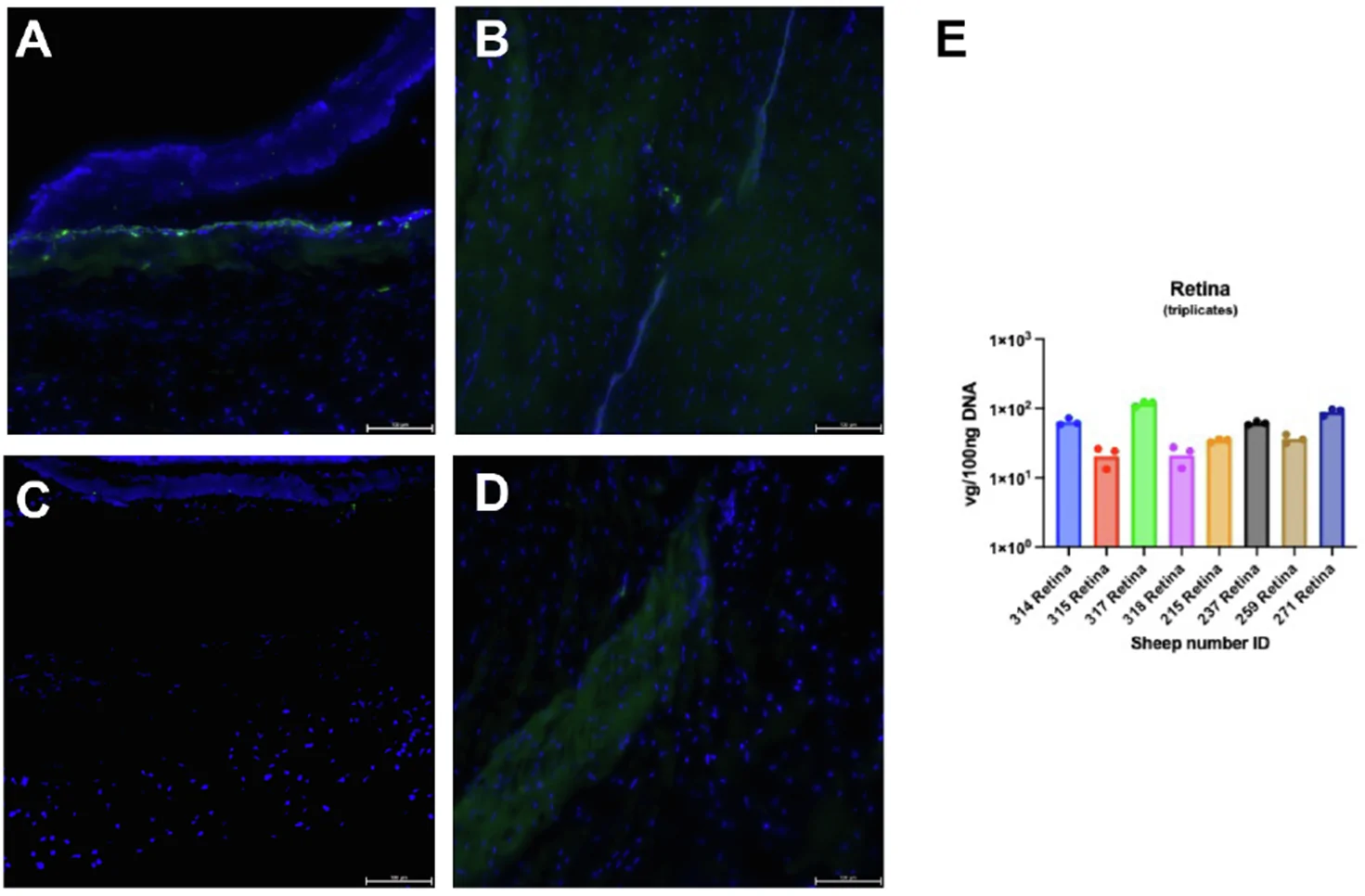
Short- and long-term therapy-treated sheep show detectable but minimal AAV9 expression in the optic nerve and retina RNAscope optimization results (A–D). The green stain represents the control probe mRNA, while the blue staining represents DAPI nuclear counterstain. (A and B) Optic disk sections stained with control probe. (C and D) Optic disk sections stained with the negative control probe. (E) Minimal vector genome expression was found in the retina of CSF-treated sheep. Scale bar, 100 μm.

Vector distribution in the retina was quantified in eight CSF-treated sheep (short and long term; *n* = 4 in each group). The retinal qPCR yielded minimal AAV9 vector genomes per sample, averaging 56 copies per 100 ng of genomic DNA (median 50; range 13–123). No amplification was observed in no-template water controls, which remained below the assay limit of detection (Figure 5).

RNAscope analysis was performed on the optic disk of six CSF-treated sheep (short and long term; *n* = 2 and *n* = 4, respectively). Consistent with vector distribution findings, RNAscope *in situ* hybridization revealed only faint fluorescence in the region of the optic disk in treated sheep, indicating minimal, but detectable, AAV9 vector signal in the optic disk. No labeling was detected in negative controls (Figure 5). Lastly, HexA activity in the retina was similar across all groups (Table S1).

## DISCUSSION

The current report expands on our knowledge of retinal abnormalities in TSD. The goals of this study were to determine retinal and optic nerve lesions in a Jacob sheep model of TSD and to establish whether gene therapy delivered intravenously or via the CSF improves neuroinflammation and retinal pathology. As we expected, untreated TSD and AAV9-treated TSD sheep eyes were grossly normal, with no overt abnormalities on retinal dissection and normal anterior segments. Microscopic evaluation demonstrated that the lesions in sheep are limited to the inner retina and optic nerve. The RGCs are engorged with microvesicles confirmed to be GM2 with extensive inner retinal astrocytosis and microgliosis. The optic nerve of untreated TSD and IV-treated TSD sheep showed extensive axonal loss and degeneration. RGC density was decreased in TSD and IV-treated sheep with significant GM2 storage. Short-term CSF therapy in sheep, but not long term, improved retinal pathology. Minimal retinal transduction and transgene expression were also observed.

In human patients with TSD, RGCs and cerebral cortical neurons demonstrate similar lesions.^27^ Previously, it was unclear whether abnormalities within the brain and spinal cord might also be present in RGCs. Veterinary literature is limited to a single case series where four TSD sheep had widespread neuronal lesions, and a single TSD sheep had retinal histopathology with storage material distending the cytoplasm of RGCs.^34^ Human ocular pathology reports are similarly limited and composed primarily of case reports.^10,24-27,35,36^ In humans, a cherry-red spot is seen in infantile forms but is inconsistent in juvenile subtypes, in which retinitis pigmentosa and optic nerve atrophy can be seen late in the disease course.^8^ The Jacob sheep TSD model recapitulates neurologic features similar to juvenile and late-onset TSD patients.^37^ Furthermore, retinal thickness measurements and photoreceptor morphology were normal in TSD sheep, suggesting photoreceptor degeneration is not a feature, although significant optic nerve degeneration is present.

Total retinal thickness measurements were normal across all groups in two regions, but that may not reflect the global retinal status. Additionally, the RPE is involved in multiple veterinary storage diseases with variable effects on photoreceptor function.^38-42^ In Batten disease, the RPE is relatively spared in the sheep model with widespread retinal degeneration, and, in GM2-deficient mice, GM2 does not accumulate in the outer retina, indicating the RPE is not the primary storage site.^38,43^ In the present study, RPE thickness did not change in peripheral and midcentral regions. Despite normal photoreceptor thickness across all groups, all sheep showed evidence of RPE vacuolization of unknown significance. Thus, further ultrastructural evaluation of the RPE would be required to further interrogate early metabolic/storage changes within the RPE.^44^

Optic nerve involvement in metabolic disorders, particularly lysosomal storage diseases, often results from apoptosis of the RGCs, oligodendrocytes, or the supporting vascular system.^1^ Optic nerve degeneration can be seen in TSD with numerous spheroids. Spheroids or axonal swellings occur in many CNS neurogenetic diseases and lysosomal storage disorders^45-48^ and contribute to marked functional impairment.^49-51^ Demyelination of the optic nerve was also demonstrated in TSD sheep, which can lead to optic nerve atrophy, as described in multiple lysosomal storage diseases.^52^ This pathology reduces vision, as observed in infantile TSD patients by 12–18 months of age, progressing to blindness by 30 months.^9^ We estimated the optic nerve axon count in normal adult sheep to be ~700,000 axons and demonstrated that TSD causes severe optic nerve degeneration, with 40% of axons lost compared with controls. The short-term CSF therapy partially prevented optic nerve axon loss and significantly reduced optic nerve damage. Direct comparisons of IV vs. CSF therapy cannot be made, as a short-term IV therapy group was not evaluated. Additionally, CSF therapy sheep received twice as much vector as IV-treated sheep. Optic nerve abnormalities in TSD are primarily due to RGC apoptosis, with possible oligodendrocyte loss.^37^ Further investigation into the duration of optic nerve preservation is required, as intravitreal injection, repeated injections, or increased dosages may be necessary.

Retinal histology in humans with TSD shows relatively normal retinal morphology, except for swollen RGCs containing PAS-positive material and eccentric nuclei.^25,27^ Similarly, untreated TSD sheep exhibit normal retinal layering with variably staining PAS-positive material in RGCs. Lipid accumulation of amacrine cells of the INL, with sparing of horizontal, bipolar, and photoreceptor cells, has been described in a human patient.^26^ We demonstrated lipid accumulation of GM2 in RBPMS-negative cells, suggesting that GM2 may be accumulating in amacrine cells of this TSD sheep model. Furthermore, we report GM2 immunolabeling in TSD in RGCs for the first time using RBPMS. Storage of GM2 in RGCs has previously been assumed but never confirmed with immunohistochemistry. Mirroring optic nerve data, RGC density, GM2 volume within RGCs, and GM2 signal intensity were rescued with short-term CSF therapy. However, these calculations are local approximations ventral to the optic nerve to avoid superimposition; regional estimates could influence interpretations, as small sample areas do not serve as global proxies for retinal status. We also appreciated more GM2-positive, RBPMS-negative cells in the GCL of TSD sheep. While we speculate that these are amacrine cells, they could also be nonviable RGCs; further investigation is required.

We hypothesize that the transient therapeutic effects of CSF-delivered AAV9 result from partial RGC transduction, producing early, localized correction that temporarily stabilizes retinal function. With disease progression, non-transduced RGCs continue to degenerate and drive a progressive neuroinflammatory response, which creates a toxic microenvironment that eventually overwhelms the initially corrected cells. Incomplete cellular targeting and secondary neuroinflammatory propagation may underlie the loss of long-term efficacy, as short-term-treated sheep showed inflammatory populations similar to those observed in long-term-treated sheep. Therefore, enhancing RGC coverage or mitigating chronic inflammation could prolong therapeutic benefits.

Across studies, retinal transduction varies following different routes and subtypes of AAV therapy, underscoring the need for further optimization. Currently, Luxturna (voretigene neparvovec-rzyl) is the only US Food and Drug Administration (FDA)-approved AAV-based gene therapy for inherited retinal disease.^53^ With regard to TSD sheep, thalamic and intracerebroventricular injections of AAVrh8 in TSD sheep led to a delayed onset of symptoms with enhanced quality of life and vision retention.^15^ AAV gene transfer to the CNS using a self-complementary AAV9 vector expressing GFP was also examined in sheep. CSF delivery to the cisterna magna and lumbar space led to widespread vector distribution, although no genome copies were detected in the retina following cisterna magna injection.^54^ Thus, CSF delivery of AAV gene therapy is a sufficient approach for correcting CNS abnormalities in TSD Jacob sheep; however, correction of ocular lesions is partial and incomplete following CSF delivery. More localized administration, such as intravitreal injection, is likely required.

Intracerebroventricular or cisterna magna injections are suspected to result in greater retinal transduction than intrathecal injections, with serotype likely playing a role. In mice, intrathecal administration of AAV9 has been shown to transduce the retina despite a mature blood ocular barrier.^55^ AAV9 also transduced the retina in neonatal mice with systemic delivery.^56-60^ Low ocular transduction of AAV9 was observed in rhesus macaques following cisterna magna injection,^61^ but pan-retinal transduction was observed with intravenous, intravitreal, and subretinal delivery in mice.^62^ Furthermore, intravitreal injection of AAVrh8 and AAVrh10 in mice transduced RGCs, amacrine cells, and bipolar cells.^63^ Clearly, studies are mixed and variable, highlighting the importance of future research on intravitreal or subretinal injections in TSD sheep to determine whether efficacy is increased.

The mechanism by which AAV9-Bic_HexA/HexB CSF therapy corrects the retina remains undefined, with minimal transduction and transient therapeutic benefit observed with vector genomes and RNA scope. Despite transient optic nerve and RGC improvements with CSF therapy, retinal HexA activity did not differ across groups. This could be attributed to the limitations of whole-retina homogenate analysis, in which localized enzymatic changes within RGCs may be diluted below detection thresholds. Previously, the optic nerve of CSF-treated sheep has shown increased, but non-significant, Hex A activity versus that of untreated TSD sheep.^23^ GM2 ganglioside levels in peripheral nerves remained unchanged in untreated TSD, IV, and short-term CSF therapy sheep, except in the optic nerve. Additionally, GM2 storage and immunofluorescence data in the thalamus indicate dramatic but incomplete improvement.^23^ Given that the lateral geniculate nucleus (LGN) resides within the caudal thalamus, we hypothesize that intracerebroventricular (ICV) injection transduces the LGN. From the LGN, AAV9 or HexA travels retrograde along the optic tract to the optic nerve and retina. Retrograde axonal transport of AAV9 or HexA, and/or diffusion via CSF, likely play dual roles. Transduction may occur along optic nerve axons through CSF-mediated exposure at the optic nerve or chiasm, as these regions are associated with sufficient CSF spaces.^64^ However, the beneficial effect is lost in the long term. Since HexA expression in the thalamus does not change between short- and long-term groups, it seems less likely that HexA cross-correction is responsible. The effect may be due to a loss of transduced cell turnover that projects to the eye or within the ocular compartment and or the optic nerve. In aggregate, these findings indicate that direct targeting via intravitreal injection may be needed to achieve longer-term RGC efficacy.

Retinal transduction after CSF delivery of AAV has been documented in murine models for Batten’s disease and MPS IIIa following ICV injection.^65,66^ This is the first study to demonstrate retinal and optic nerve improvements following CSF-delivered AAV9 in a large-animal model. Further ocular *in vivo* studies in sheep with TSD are required. Following affected and treated TSD sheep over time using electroretinography (ERG), optical coherence tomography (OCT), fluorescein angiography, and serial fundus examinations may further advance understanding of this disease. An accurate understanding of ocular lesions, particularly retinal and optic nerve changes, is essential. Amelioration of ophthalmic histopathologic findings would be a crucial marker in quantifying treatment success. Non-invasive tools such as ERG and OCT could help monitor disease progression. Ultimately, understanding ocular changes associated with TSD may provide clinicians with a deeper understanding of visual pathology in their patients.

This study had several limitations: first, because it was not a prospective study, control sheep were not age or sex matched, which could confound the results. However, despite age differences, TSD sheep show significant abnormalities early in life (3 months of age), and the ocular lesions are likely captured in our cohorts, as by 3 months of age, GM2 is significantly increased in the CSF with widespread cerebral demyelination in TSD sheep.^37^ The increased age of IV-treated sheep may impart more significant abnormalities, with a possibility that the therapeutic benefits of IV-treatment were understated. AAV9-Bic_HexA/HexB CSF therapy ameliorated TSD lesions in the short term, suggesting that this therapy may reach the eye, particularly the optic nerve, but lacks long-term efficacy for addressing ophthalmic lesions, whereas IV administration did not. Although the reason this effect was lost years later remains unclear, neuroinflammation is a likely mechanism. Importantly, although the findings indicate a temporary, incomplete recovery of optic nerve pathology, the ocular lesions in TSD will likely be receptive to more appropriate future gene-therapy delivery methods and may result in further improvements.

## MATERIALS AND METHODS

### Vector details and dose

The AAV9-Bic_HexA/HexB bidirectional vector was manufactured by triple transfection and purified by iodixanol gradient ultracentrifugation as previously described,^22^ except for substitution with species-specific ovine wild-type cDNAs for Hex α (*HEXA*) and Hex β (*HEXB*). The final formulation was in Dulbecco’s PBS without magnesium and calcium (Thermo Fischer Scientific, Waltham, MA, USA). Our bicistronic AAV9 vector carries an expression cassette that is composed of a central cytomegalovirus (CMV) enhancer that is flanked by two chicken beta-actin promoters in opposite directions without introns. *HEXA* is flanked by an SV40 poly(A) signal, whereas the sheep *HEXB* is flanked by an rabbit beta-globin (RBG) poly(A) signal.

### Animals and surgery

The animal procedures were approved by Auburn University, Cummings School of Veterinary Medicine at Tufts University, and UMass Chan Medical School Institutional Animal Care and Use Committees. TSD-affected sheep were randomly assigned into groups (untreated, IV therapy, CSF therapy).

The sheep cohort whose eyes were analyzed consisted of normal (*n* = 3), untreated TSD (*n* = 4), IV AAV9-Bic_HexA/HexB treated (*n* = 3), and CSF AAV9-Bic_HexA/HexB treated with intracerebroventricular, cisterna magna, and lumbar intrathecal administration; *n* = 7 (*n* = 3 short-term, *n* = 4 long-term) (Figure S10; Table S2). Two additional TSD sheep (a 7-month-old male and a 9-month-old female) had whole globes available for initial analysis. These two sheep were used for a pilot evaluation of retinal lesions and were included only in histopathologic assessment, not in immunohistochemical analysis. Normal control sheep included two 13-month-old crossbred sheep (one male and one female) and one 6-year-old female Jacob sheep. Untreated TSD consisted of a 7.5-month-old male, an 8-month-old female, a 9-month-old female, and a 13-month-old female. Intravenous therapy sheep (all female) were treated with 5 × 10^13^ vector genomes (vg) per kg at 11 (*n* = 1) and 13 (*n* = 2) days of age with humane endpoints of 16.3, 21.7, and 23.7 months. Short-term CSF therapy sheep (two males, one female) were treated with 1 × 10^14^ vg/kg at 23, 24, and 31 days of age with endpoints of 5 months (*n* = 3). Long-term CSF sheep (two males, two females) were treated with 1 × 10^14^ vg/kg at 25, 32, 35, and 40 days of age with endpoints of 10, 45, 47, and 49 months. Treated Jacob sheep were sedated with 0.1 mg/kg midazolam, and intravenous jugular catheters were placed. Intravenously treated sheep received a bolus injection of AAV vectors to the jugular vein; CSF-treated sheep received 75% of the dose through bilateral ICV, 12.5% through the cisterna magna, and 12.5% through the lumbar intrathecal injection.

The TSD-affected sheep were humanely euthanized by pentobarbital (150 mg/kg) overdose. After euthanasia, TSD untreated and treated globes were removed and stored in 4% paraformaldehyde (PFA) within 2 h of euthanasia. In all sheep eyes, a stab incision 2 mm posterior to the dorsal limbus was made to ensure intraocular infusion of fixative. For control eyes, globes were removed and stored in 4% PFA within 48 h. Control eyes were obtained from a local meat farm (Superior Farms, CA) (*n* = 2, 13-month-old crossbred sheep) or deceased due to natural causes (*n* = 1, 6-year-old Jacob sheep). As the original intention for TSD untreated and treated globes was not for ocular analysis, storage times in 4% PFA varied and were 1 week (*n* = 2) and 2–3 years (*n* = 2) for TSD untreated sheep, 2 years for TSD IV-treated (*n* = 3), 1 year (*n* = 3) and 5 years (*n* = 1) for short-term CSF therapy-treated sheep, and 1 year (*n* = 2) and 3 years (*n* = 1) for long-term CSF therapy-treated sheep. Control eyes were stored in 4% PFA for less than 1 week. Retinas were dissected, and two 8 × 8-mm quadrants were harvested for each sheep from the superotemporal retina. Retinal-thickness measurements were performed on the peripheral retina (superotemporal superior; all cohorts) sections and midcentral retina (superotemporal inferior; controls, TSD, IV, short-term CSF), whereas histopathology and immunohistochemistry were performed on the midcentral retina (superotemporal inferior). Four-millimeter retinal punch biopsies were taken ventral to the optic nerve for retinal whole mounts, stored in 100% methanol at −80°C, and rehydrated before analysis.

### Optic nerve analysis

The ON was transected 2 mm posterior to the sclera (cut perpendicular to the longitudinal axis) and stored in 4% PFA. Samples were transferred to 6% glutaraldehyde. A 500-μm section was removed using an ultramicrotome (EM UC6, Leica), and the sections were then post-fixed with 4% osmium tetroxide and embedded in epoxy resin. Cross sections of the ON, 1 μm thick, were cut using an ultramicrotome (EM UC6, Leica), stained with PPD, and mounted on glass slides.

Optic nerve PPD-stained images were acquired at 100× under an oil emersion objective (BZ-X800, Keyence). Eight percent of the optic nerve area was imaged per sample to avoid the axon-count variability observed when sampled areas were less than 8%. Optic nerve myelinated axon counts were performed manually using ImageJ (version 1.54i 03) cell counter. Quantitative and qualitative analyses of optic nerve sections were performed on 8% of the total optic nerve area.^67^ Qualitative analysis of axon lesions was performed using the criteria of optic nerve damage scores on PPD-stained myelinated axons by two masked graders (B.D.S., S.A.A.) with experience in axon morphology.^68^ Briefly, the criteria of optic nerve damage scores proposed by Pang and Clark score ONs from 1 to 5, with a score of 1 indicating no gliosis or axonal swelling with few dark staining axons, whereas a score of 5 indicates <10% of axons are alive with marked gliosis and darkly stained axons making up 95% of the ON.

### Histology and immunohistochemistry

Thirteen-month-old, crossbred sheep were used for antibody optimization. PFA (4%)-stored retinal sections were submitted to the Comparative Ocular Pathology Laboratory of Wisconsin for routine processing, paraffin embedding, and sectioning. Retinal sections were routinely stained with hematoxylin and eosin (H&E) and PAS. LFB staining was performed on a subset of samples. Unstained sections were sent to University of California (UC) Davis for immunohistochemical evaluation.

Paraffin-embedded retinal sections were dewaxed with xylene, rehydrated through an alcohol series, and washed. Antigen retrieval was performed with 10 mM citric acid and 0.05% Tween for 30 min with a pH of 6.0. Sections were washed with PBS and 0.1% Triton X-100 with 0.1% bovine serum albumin. Blocking was performed with 10% normal goat serum (NGS) for 30 min and subsequently incubated overnight at 4°C with one of the following primary antibodies at 1:500 dilution: anti-RBPMS(rabbit polyclonal, GTX118619, Genetex, Irvine, CA), anti-GM2 (mouse monoclonal, A2576, TCI America, Portland, OR), anti-GFAP (mouse monoclonal, G3893, Sigma, St. Louis, MO), and anti-IBA1 (rabbit polyclonal, 019-19741, Wako Chemicals, Richmond, VA). After washing, the appropriate secondary antibody, filtered using a 0.2-μm syringe filter, was applied for 1 h at 1:500 dilution: Alexa Fluor 488 anti-mouse IgG (A-11029, Invitrogen, Waltham, MA) and Cy3 anti-rabbit IgG (111-165-144, Jackson ImmunoResearch, West Grove, PA). Nuclei were then stained with DAPI. To validate the specificity of antigen-antibody reactions, secondary antibody was used alone as a negative control. Photography was performed using a fluorescent confocal microscope (Fluoview FV300, Olympus). For all images, the detector sensitivity (high voltage [HV]) was adjusted manually to below saturation with gain set to 1.0 and offset to 3%.

### Retinal whole mounts

Four-millimeter retinal punches immediately inferior to the optic disk were stored in 100% methanol at −80°C. The inferior retina in sheep contains a lower RGC density versus other quadrants and was chosen to allow accurate counting in an attempt to avoid superimposition of RGCs.^69^ Samples were rehydrated in sequential 66/33/0% methanol in PBS (1×) solutions. Blocking was performed for 3 h at 4°C using 5% NGS with 0.3% Triton X-100 to create antibody diluting solution (ADS). Whole mounts were incubated with primary antibody in ADS for 48 h at 4°C. Samples were washed in ADS prior to incubation with filtered secondary antibody in ADS for 24 h at 4°C. Samples were again washed with ADS and DAPI followed by a series of PBS (1×) washes including overnight washing. The following day, whole mounts were air dried and mounted on glass slides for analysis. For the detection of GM2 ganglioside, mouse anti-GM2 monoclonal IgM antibody (1:500; TCI America) was used as a primary antibody and Cy2 goat anti-mouse IgM (1:500; Jackson ImmunoResearch) was used as a secondary antibody.

Confocal analysis was performed (Fluoview FV300, Olympus). z stack images were acquired on 20× magnification using a 1-μm z thickness, capturing the entirety of the retinal GCL. HV was set just below saturation. The gain was set to 1% and offset to 10% for DAPI and 15% for GM2 and RBPMS channels. Setting parameters were kept uniform for acquisition of all images. Five random images avoiding whole-mount edges were taken per sample for RGC quantification based on RBPMS immunolabeling at 20× magnification. ImageJ (version 1.54i 03) cell-counter tool was used to quantify RGCs per sample.

RGC surfaces were automatically created using image analysis software (Imaris 9, Oxford Instruments). Specifically, surfaces were created for RBPMS and GM2 fluorescence. Smooth detail was set to 0.5 μm. Thresholds were set at 300 for RBPMS and GM2. Images were filtered by number of voxels (12,000 for RBPMS; 6,500 for GM2) with background subtraction of 10 μm. GM2 and RBPMS volume and signal intensity were automatically calculated using Imaris 9 software.

### Biodistribution analysis of vector genomes

Total DNA extraction from retina tissues samples was performed by using a DNeasy blood and tissue kit (QIAGEN, Hilden, Germany, catalog #69506). Vector genome content in each sample was determined using 100 ng of total DNA by the qPCR method using primers and probes for the SV40 poly(A) cassette in the vector. 6-carboxyl-fluorescein (FAM) detection was measured in a Bio-Rad (CFX Opus 96) qPCR machine. The lower limit of detection in the qPCR assay was 100 genome copies/100 ng of total DNA. The number of vector genomes was plotted in GraphPad PRISM 8.0 (GraphPad Software, San Diego, CA).

### *In situ* hybridization (RNAscope) and image acquisition

Tissue was harvested, embedded in OCT, and frozen; OCT-embedded blocks were cut into 10-μm sections. Sample preparation for RNAscope staining was performed following the manufacturer’s guidelines. An RNAscope probe was designed (RNAscope Probe: BiCb6-No-XOaBt-C1) to target the CMV region sequence in the AAV9 HexA/HexB bicistronic expression cassette. RNAscope Negative Control Probe: DapB (catalog 310043) was used as a negative control. *In situ* hybridization to detect probe signal was performed using RNAscope Multiplex Fluorescent Reagent Kit v2 (catalog 323100) and TSA Vivid Fluorophore 520 (PN 323271) following the manufacturer’s instructions. Fluorescence was captured with a Leica DMi8 Thunder imager fluorescent microscope (Leica Microsystems, Buffalo Grove, IL) at 20× and 40× magnifications. Images were analyzed with LAS X software.

### Hexosaminidase activity

Whole retinal sections were flash frozen in liquid nitrogen, and HexA activity (nmol/h/mg) was measured using a 4MU fluorogenic substrate (4MU-6-sulfo-2-acetamido-2-deoxy-β-D-glucopyranoside [MUGS]). Fluorescence was quantified against a 4MU standard curve, and enzyme activity was normalized to protein concentration as determined by the Bradford assay.

### Statistics

Statistics were calculated using GraphPad PRISM (10.0.2.). Descriptive statistics were used. Histological and immunohistochemical data were compared and analyzed using an unpaired *t* test as well as mean ± standard deviation and one-way ANOVA. Categorical data were represented as median and range. Significance was set at a two-tailed *p* value of 0.05.

## Supplementary Material

S1. Figures S1–S10 and Tables S1 and S2.

Supplemental information can be found online at https://doi.org/10.1016/j.ymthe.2026.06.018.

## References

[1] Maegawa GHB, Stockley T, Tropak M, Banwell B, Blaser S, Kok F, Giugliani R, Mahuran D, and Clarke JTR. (2006). The natural history of juvenile or subacute GM2 gangliosidosis: 21 new cases and literature review of 134 previously reported. Pediatrics 118, e1550–e1562. 17015493 10.1542/peds.2006-0588 PMC2910078

[2] Neudorfer O, Pastores GM, Zeng BJ, Gianutsos J, Zaroff CM, and Kolodny EH. (2005). Late-onset Tay-Sachs disease: phenotypic characterization and genotypic correlations in 21 affected patients. Genet. Med. 7, 119–123. 15714079 10.1097/01.gim.0000154300.84107.75

[3] Sango K, Yamanaka S, Hoffmann A, Okuda Y, Grinberg A, Westphal H, McDonald MP, Crawley JN, Sandhoff K, Suzuki K, and Proia RL. (1995). Mouse models of Tay-Sachs and Sandhoff diseases differ in neurologic phenotype and ganglioside metabolism. Nat. Genet. 11, 170–176. 7550345 10.1038/ng1095-170

[4] Torres PA, Zeng BJ, Porter BF, Alroy J, Horak F, Horak J, and Kolodny EH. (2010). Tay-Sachs disease in Jacob sheep. Mol. Genet. Metab. 101, 357–363. 20817517 10.1016/j.ymgme.2010.08.006

[5] Kuil LE, López Martí A, Carreras Mascaro A, van den Bosch JC, van den Berg P, van der Linde HC, Schoonderwoerd K, Ruijter GJG, and van Ham TJ. (2019). Hexb enzyme deficiency leads to lysosomal abnormalities in radial glia and microglia in zebrafish brain development. Glia 67, 1705–1718. 31140649 10.1002/glia.23641 PMC6772114

[6] Cork LC, Munnell JF, Lorenz MD, Murphy JV, Baker HJ, and Rattazzi MC. (1977). GM2 ganglioside lysosomal storage disease in cats with beta-hexosaminidase deficiency. Science 196, 1014–1017. 404709 10.1126/science.404709

[7] Seyrantepe V, Demir SA, Timur ZK, Von Gerichten J, Marsching C, Erdemli E, Oztas E, Takahashi K, Yamaguchi K, Ates N, (2018). Murine Sialidase Neu3 facilitates GM2 degradation and bypass in mouse model of Tay-Sachs disease. Exp. Neurol. 299, 26–41. 28974375 10.1016/j.expneurol.2017.09.012

[8] Udwadia-Hegde A, and Hajirnis O. (2017). Temporary Efficacy of Pyrimethamine in Juvenile-Onset Tay-Sachs Disease Caused by 2 Unreported HEXA Mutations in the Indian Population. Child. Neurol. Open 4, 2329048X16687887. 10.1177/2329048X16687887 PMC5417282 28503624

[9] Ramani PK, and Parayil Sankaran B. (2021). Tay-Sachs Disease. In StatPearls (Treasure Island (FL). 33232090

[10] Jabbehdari S, Jafari N, Golnik K, Shahriari M, and Karimzadeh P. (2018). Ophthalmologic Findings in Patients with Neuro-metabolic Disorders. J. Ophthalmic Vis. Res. 13, 34–38. 29403587 10.4103/jovr.jovr_242_16 PMC5782454

[11] Solovyeva VV, Shaimardanova AA, Chulpanova DS, Kitaeva KV, Chakrabarti L, and Rizvanov AA. (2018). New Approaches to Tay-Sachs Disease Therapy. Front. Physiol. 9, 1663. 30524313 10.3389/fphys.2018.01663 PMC6256099

[12] Bainbridge JWB, Smith AJ, Barker SS, Robbie S, Henderson R, Balaggan K, Viswanathan A, Holder GE, Stockman A, Tyler N, (2008). Effect of gene therapy on visual function in Leber’s congenital amaurosis. N. Engl. J. Med. 358, 2231–2239. 18441371 10.1056/NEJMoa0802268

[13] Cideciyan AV, Aleman TS, Boye SL, Schwartz SB, Kaushal S, Roman AJ, Pang JJ, Sumaroka A, Windsor EAM, Wilson JM, (2008). Human gene therapy for RPE65 isomerase deficiency activates the retinoid cycle of vision but with slow rod kinetics. Proc. Natl. Acad. Sci. USA 105, 15112–15117. 18809924 10.1073/pnas.0807027105 PMC2567501

[14] Hauswirth WW, Aleman TS, Kaushal S, Cideciyan AV, Schwartz SB, Wang L, Conlon TJ, Boye SL, Flotte TR, Byrne BJ, and Jacobson SG. (2008). Treatment of leber congenital amaurosis due to RPE65 mutations by ocular subretinal injection of adeno-associated virus gene vector: short-term results of a phase I trial. Hum. Gene Ther. 19, 979–990. 18774912 10.1089/hum.2008.107 PMC2940541

[15] Gray-Edwards HL, Randle AN, Maitland SA, Benatti HR, Hubbard SM, Canning PF, Vogel MB, Brunson BL, Hwang M, Ellis LE, (2018). Adeno-Associated Virus Gene Therapy in a Sheep Model of Tay-Sachs Disease. Hum. Gene Ther. 29, 312–326. 28922945 10.1089/hum.2017.163

[16] Bradbury AM, Peterson TA, Gross AL, Wells SZ, McCurdy VJ, Wolfe KG, Dennis JC, Brunson BL, Gray-Edwards H, Randle AN, (2017). AAV-mediated gene delivery attenuates neuroinflammation in feline Sandhoff disease. Neuroscience 340, 117–125. 27793778 10.1016/j.neuroscience.2016.10.047 PMC5154837

[17] Bradbury AM, Gray-Edwards HL, Shirley JL, McCurdy VJ, Colaco AN, Randle AN, Christopherson PW, Bird AC, Johnson AK, Wilson DU, (2015). Biomarkers for disease progression and AAV therapeutic efficacy in feline Sandhoff disease. Exp. Neurol. 263, 102–112. 25284324 10.1016/j.expneurol.2014.09.020 PMC4262540

[18] Bradbury AM, Cochran JN, McCurdy VJ, Johnson AK, Brunson BL, Gray-Edwards H, Leroy SG, Hwang M, Randle AN, Jackson LS, (2013). Therapeutic response in feline sandhoff disease despite immunity to intracranial gene therapy. Mol. Ther. 21, 1306–1315. 23689599 10.1038/mt.2013.86 PMC3702100

[19] McCurdy VJ, Rockwell HE, Arthur JR, Bradbury AM, Johnson AK, Randle AN, Brunson BL, Hwang M, Gray-Edwards HL, Morrison NE, (2015). Widespread correction of central nervous system disease after intracranial gene therapy in a feline model of Sandhoff disease. Gene Ther. 22, 181–189. 25474439 10.1038/gt.2014.108

[20] Eichler F, Cataltepe OI, Daci R, Puri AS, Taghian T, Jiang X, Shazeeb MS, Kuhn A, Hader A, Celik H, (2025). Dual-vector rAAVrh8 gene therapy for GM2 gangliosidosis: a phase 1/2 trial. Nat. Med. 31, 2927–2935. 40817303 10.1038/s41591-025-03822-4 PMC12443631

[21] Flotte TR, Cataltepe O, Puri A, Batista AR, Moser R, McKenna-Yasek D, Douthwright C, Gernoux G, Blackwood M, Mueller C, (2022). AAV gene therapy for Tay-Sachs disease. Nat. Med. 28, 251–259. 35145305 10.1038/s41591-021-01664-4 PMC10786171

[22] Lahey HG, Webber CJ, Golebiowski D, Izzo CM, Horn E, Taghian T, Rodriguez P, Batista AR, Ellis LE, Hwang M, (2020). Pronounced Therapeutic Benefit of a Single Bidirectional AAV Vector Administered Systemically in Sandhoff Mice. Mol. Ther. 28, 2150–2160. 32592687 10.1016/j.ymthe.2020.06.021 PMC7544971

[23] Taghian T, Gallagher J, Bertrand S, Baker WC, Lopez Mercado K, Benatti HR, Hall E, Lopez Y, McElroy A, McCarthy JT, (2025). Five-year analysis of efficacy and safety of a bidirectional AAV gene therapy in Tay-Sachs sheep. J. Clin. Invest. 135, e182942. 10.1172/JCI182942. 41026525 PMC12646665

[24] Cogan DG, Kuwabara T, Kolodny E, and Driscoll S. (1984). Gangliosidoses and the fetal retina. Ophthalmology 91, 508–512. 6330639 10.1016/s0161-6420(84)34274-9

[25] Yanoff MS Ocular Pathology. Seventh Edition, 407–410 (2014).

[26] Nagashima K, Kukichi F, Suzuki Y, and Abe T. (1981). Retinal amacrine cell involvement in Tay-Sachs disease. Acta Neuropathol. 53, 333–336. 7223376 10.1007/BF00690376

[27] Harcourt RB, and Dobbs RH. (1968). Ultrastructure of the retina in Tay-Sach’s disease. Br. J. Ophthalmol. 52, 898–902. 5700673 10.1136/bjo.52.12.898 PMC506712

[28] Müller G, Alldinger S, Moritz A, Zurbriggen A, Kirchhof N, Sewell A, and Baumgärtner W. (2001). GM1-gangliosidosis in Alaskan huskies: clinical and pathologic findings. Vet. Pathol. 38, 281–290. 11355658 10.1354/vp.38-3-281

[29] Alroy J, Orgad U, DeGasperi R, Richard R, Warren CD, Knowles K, Thalhammer JG, and Raghavan SS. (1992). Canine GM1-gangliosidosis. A clinical, morphologic, histochemical, and biochemical comparison of two different models. Am. J. Pathol. 140, 675–689. 1546746 PMC1886155

[30] Fox J, Li YT, Dawson G, Alleman A, Johnsrude J, Schumacher J, and Homer B. (1999). Naturally occurring GM2 gangliosidosis in two Muntjak deer with pathological and biochemical features of human classical Tay-Sachs disease (type B GM2 gangliosidosis). Acta Neuropathol. 97, 57–62. 9930895 10.1007/s004010050955

[31] Kolicheski A, Johnson GS, Villani NA, O’Brien DP, Mhlanga-Mutangadura T, Wenger DA, Mikoloski K, Eagleson JS, Taylor JF, Schnabel RD, and Katz M. (2017). GM2 Gangliosidosis in Shiba Inu Dogs with an In-Frame Deletion in HEXB. J. Vet. Intern. Med. 31, 1520–1526. 28833537 10.1111/jvim.14794 PMC5598891

[32] Fishelson L, Delarea Y, and Galil BS. (2000). Pathological alterations typical of human Tay-Sachs disease, in the retina of a deep-sea fish. Naturwissenschaften 87, 363–365. 11013889 10.1007/s001140050741

[33] Herron MA, Martin JE, and Joyce JR. (1978). Quantitative study of the decussating optic axons in the pony, cow, sheep, and pig. Am. J. Vet. Res. 39, 1137–1139. 677532

[34] Porter BF, Lewis BC, Edwards JF, Alroy J, Zeng BJ, Torres PA, Bretzlaff KN, and Kolodny EH. (2011). Pathology of GM2 gangliosidosis in Jacob sheep. Vet. Pathol. 48, 807–813. 21123862 10.1177/0300985810388522

[35] Kivlin JD, Sanborn GE, and Myers GG. (1985). The cherry-red spot in Tay-Sachs and other storage diseases. Ann. Neurol. 17, 356–360. 4004157 10.1002/ana.410170409

[36] Garner A. (1973). Ocular pathology of GM2 gangliosidosis–Type 2 (Sandhoff’s disease). Br. J. Ophthalmol. 57, 514–520. 4353593 10.1136/bjo.57.7.514 PMC1214963

[37] Story B, Taghian T, Gallagher J, Koehler J, Taylor A, Randle A, Nielsen K, Gross A, Maguire A, Carl S, (2021). Natural history of Tay-Sachs disease in sheep. Mol. Genet. Metab. 134, 164–174. 34456134 10.1016/j.ymgme.2021.08.009 PMC8811770

[38] Murray SJ, and Mitchell NL. (2022). Natural history of retinal degeneration in ovine models of CLN5 and CLN6 neuronal ceroid lipofuscinoses. Sci. Rep. 12, 3670. 35256654 10.1038/s41598-022-07612-7 PMC8901734

[39] Narfström K, Wrigstad A, Ekesten B, and Berg A-. (2007). Neuronal ceroid lipofuscinosis: clinical and morphologic findings in nine affected Polish Owczarek Nizinny (PON) dogs. Vet. Ophthalmol. 10, 111–120. 17324167 10.1111/j.1463-5224.2007.00527.x

[40] Goebel HH, and Dahme E. (1986). ULTRASTRUCTURE OF RETINAL PIGMENT EPITHELIAL AND NEURAL CELLS IN THE NEURONAL CEROID-LIPOFUSCINOSIS AFFECTED DALMATIAN DOG. Retina 6, 179–188. 3025985 10.1097/00006982-198600630-00009

[41] Aguirre G, Stramm L, and Haskins M. (1983). Feline mucopolysaccharidosis VI: General ocular and pigment epithelial pathology. Invest Ophthalmol. Vis. Sci. 24, 991–1007. 6409835

[42] Kick GR, Marzano SL, Ota-Kuroki J, Bullock G, and Katz ML. (2025). Retinal Function Deficits in American Staffordshire Terriers with a Late-Onset Neurodegenerative Disease Associated with an ARSG Variant. Vet. Sci. 12, 1078. 41295716 10.3390/vetsci12111078 PMC12656862

[43] Sango K, Yamanaka S, Ajiki K, Arai N, and Takano M. (2008). Involvement of Retinal Neurons and Pigment Epithelial Cells in a Murine Model of Sandhoff Disease. Ophthalmic Res. 40, 241–248. 18437034 10.1159/000127831

[44] Landowski M, Grindel S, Hao Y, Ikeda S, Bowes Rickman C, and Ikeda A. (2023). A Protocol to Evaluate and Quantify Retinal Pigmented Epithelium Pathologies in Mouse Models of Age-Related Macular Degeneration. JoVE 64927. 10.3791/64927. PMC10311451 36971449

[45] Stokin GB, Lillo C, Falzone TL, Brusch RG, Rockenstein E, Mount SL, Raman R, Davies P, Masliah E, Williams DS, and Goldstein LSB. (2005). Axonopathy and transport deficits early in the pathogenesis of Alzheimer’s disease. Science 307, 1282–1288. 15731448 10.1126/science.1105681

[46] Adalbert R, Nogradi A, Babetto E, Janeckova L, Walker SA, Kerschensteiner M, Misgeld T, and Coleman MP. (2009). Severely dystrophic axons at amyloid plaques remain continuous and connected to viable cell bodies. Brain 132, 402–416. 19059977 10.1093/brain/awn312

[47] Ohara S, Ukita Y, Ninomiya H, and Ohno K. (2004). Axonal dystrophy of dorsal root ganglion sensory neurons in a mouse model of Niemann-Pick disease type C. Exp. Neurol. 187, 289–298. 15144855 10.1016/j.expneurol.2004.03.002

[48] Oya Y, Nakayasu H, Fujita N, Suzuki K, and Suzuki K. (1998). Pathological study of mice with total deficiency of sphingolipid activator proteins (SAP knockout mice). Acta Neuropathol. 96, 29–40. 9678511 10.1007/s004010050857

[49] Coleman MP, Adalbert R, and Beirowski B. (2005). Neuroprotective strategies in MS: lessons from C57BL/Wld(S) mice. J. Neurol. Sci. 233, 133–138. 15899498 10.1016/j.jns.2005.03.028

[50] Takeuchi H, Mizuno T, Zhang G, Wang J, Kawanokuchi J, Kuno R, and Suzumura A. (2005). Neuritic beading induced by activated microglia is an early feature of neuronal dysfunction toward neuronal death by inhibition of mitochondrial respiration and axonal transport. J. Biol. Chem. 280, 10444–10454. 15640150 10.1074/jbc.M413863200

[51] Komatsu M, Wang QJ, Holstein GR, Friedrich VL, Iwata J.i., Kominami E, Chait BT, Tanaka K, and Yue Z. (2007). Essential role for autophagy protein Atg7 in the maintenance of axonal homeostasis and the prevention of axonal degeneration. Proc. Natl. Acad. Sci. USA 104, 14489–14494. 17726112 10.1073/pnas.0701311104 PMC1964831

[52] Biswas J, Nandi K, Sridharan S, and Ranjan P. (2008). Ocular manifestation of storage diseases. Curr. Opin. Ophthalmol. 19, 507–511. 18854696 10.1097/ICU.0b013e32831215c3

[53] Russell S, Bennett J, Wellman JA, Chung DC, Yu Z-F, Tillman A, Wittes J, Pappas J, Elci O, McCague S, (2017). Efficacy and safety of voretigene neparvovec (AAV2-hRPE65v2) in patients with RPE65 -mediated inherited retinal dystrophy: a randomised, controlled, open-label, phase 3 trial. The Lancet 390, 849–860. 10.1016/S0140-6736(17)31868-8 PMC5726391 28712537

[54] Taghian T, Marosfoi MG, Puri AS, Cataltepe OI, King RM, Diffie EB, Maguire AS, Martin DR, Fernau D, Batista AR, (2020). A Safe and Reliable Technique for CNS Delivery of AAV Vectors in the Cisterna Magna. Mol. Ther. 28, 411–421. 31813800 10.1016/j.ymthe.2019.11.012 PMC7002897

[55] Gray SJ, Han Z, Bailey R, Rozenberg A, Zheng M, and Mitra R. (2017). Retinal Ganglion Cell Gene Transfer Is Achieved Following Intrathecal Administration of AAV9. Invest. Ophthalmol. Vis. Sci. 58, 4099.

[56] Bostick B, Ghosh A, Yue Y, Long C, and Duan D. (2007). Systemic AAV-9 transduction in mice is influenced by animal age but not by the route of administration. Gene Ther. 14, 1605–1609. 17898796 10.1038/sj.gt.3303029

[57] Bemelmans AP, Duqué S, Rivière C, Astord S, Desrosiers M, Marais T, Sahel JA, Voit T, and Barkats M. (2013). A single intravenous AAV9 injection mediates bilateral gene transfer to the adult mouse retina. PLoS One 8, e61618. 23613884 10.1371/journal.pone.0061618 PMC3626698

[58] Byrne LC, Lin YJ, Lee T, Schaffer DV, and Flannery JG. (2015). The expression pattern of systemically injected AAV9 in the developing mouse retina is determined by age. Mol. Ther. 23, 290–296. 25224467 10.1038/mt.2014.181 PMC4445607

[59] Gruntman AM, Su L, and Flotte TR. (2017). Retro-Orbital Venous Sinus Delivery of rAAV9 Mediates High-Level Transduction of Brain and Retina Compared with Temporal Vein Delivery in Neonatal Mouse Pups. Hum. Gene Ther. 28, 228–230. 28319444 10.1089/hum.2017.037 PMC5824662

[60] Rahim AA, Wong AMS, Hoefer K, Buckley SMK, Mattar CN, Cheng SH, Chan JKY, Cooper JD, and Waddington SN. (2011). Intravenous administration of AAV2/9 to the fetal and neonatal mouse leads to differential targeting of CNS cell types and extensive transduction of the nervous system. FASEB J. 25, 3505–3518. 21746868 10.1096/fj.11-182311

[61] Hordeaux J, Ramezani A, Tuske S, Mehta N, Song C, Lynch A, Lupino K, Chichester JA, Buza EL, Dyer C, (2023). Immune transgene-dependent myocarditis in macaques after systemic administration of adeno-associated virus expressing human acid alpha-glucosidase. Front. Immunol. 14, 1094279. 37033976 10.3389/fimmu.2023.1094279 PMC10073725

[62] Palfi A, Chadderton N, Millington-Ward S, Post I, Humphries P, Kenna PF, and Farrar GJ. (2022). eB transduces both the inner and outer retina with high efficacy in mice. Mol. Ther. Methods Clin. Dev. 25, 236–249. 35474956 10.1016/j.omtm.2022.03.016 PMC9018541

[63] Giove TJ, Sena-Esteves M, and Eldred WD. (2010). Transduction of the inner mouse retina using AAVrh8 and AAVrh10 via intravitreal injection. Exp. Eye Res. 91, 652–659. 20723541 10.1016/j.exer.2010.08.011 PMC2962726

[64] Sheng J, Li Q, Liu T, and Wang X. (2022). Cerebrospinal fluid dynamics along the optic nerve. Front. Neurol. 13, 931523. 36046631 10.3389/fneur.2022.931523 PMC9420993

[65] Beard H, Winner L, Shoubridge A, Parkinson-Lawrence E, Lau AA, Mubarokah SN, Lance T-R, King B, Scott W, Snel MF, (2024). Evaluation of neuroretina following i.v. or intra-CSF AAV9 gene replacement in mice with MPS IIIA, a childhood dementia. CNS Neurosci. Ther. 30, e14919. 39123298 10.1111/cns.14919 PMC11315678

[66] White KA, Nelvagal HR, Poole TA, Lu B, Johnson TB, Davis S, Pratt MA, Brudvig J, Assis AB, Likhite S, (2021). Intracranial delivery of AAV9 gene therapy partially prevents retinal degeneration and visual deficits in CLN6-Batten disease mice. Mol. Ther. Methods Clin. Dev. 20, 497–507. 33665223 10.1016/j.omtm.2020.12.014 PMC7887332

[67] Cull G, Cioffi GA, Dong J, Homer L, and Wang L. (2003). Estimating Normal Optic Nerve Axon Numbers in Non-Human Primate Eyes. J. Glaucoma 12, 301–306. 12897574 10.1097/00061198-200308000-00003

[68] Pang IH, and Clark AF. (2007). Rodent models for glaucoma retinopathy and optic neuropathy. J. Glaucoma 16, 483–505. 17700292 10.1097/IJG.0b013e3181405d4f

[69] Shinozaki A, Hosaka Y, Imagawa T, and Uehara M. (2010). Topography of ganglion cells and photoreceptors in the sheep retina. J. Comp. Neurol. 518, 2305–2315. 20437529 10.1002/cne.22333

