# Supplementary material for: Cerebrospinal delivery of a bidirectional AAV9 vector improves optic nerve and retinal pathology in a sheep model of Tay-Sachs disease
Source: Mol Ther. Author manuscript; Available in PMC 2026 Aug 27. (PMC13508168; doi:10.1016/j.ymthe.2026.06.018)
Supplement: Supplementary file 1 [file NIHMS2203170-supplement-S1__Figures_S1_S10_and_Tables_S1_and_S2_.pdf]

YMTHE, Volume *34*

Supplemental Information

Cerebrospinal delivery of a bidirectional AAV9 vector improves optic nerve and retinal pathology in a sheep model of Tay-Sachs disease

Brett D. Story, Sara M. Thomasy, Sara A. Adelman, Toloo Taghian, Yvette Lopez, Monica Ardon, Hikaru Shiraishi, Sabina Khan, Meher Khan, Maria Do, Hector R. Benatti, Stephanie Bertrand, Rachel Prestigiacomo, Rachael Gately, Douglas R. Martin, Miguel Sena-Esteves, Jun Xie, Guangping Gao, Leandro B.C. Teixeira, Gillian C. Shaw, Nicholas Marsh-Armstrong, and Heather L. Gray-Edwards

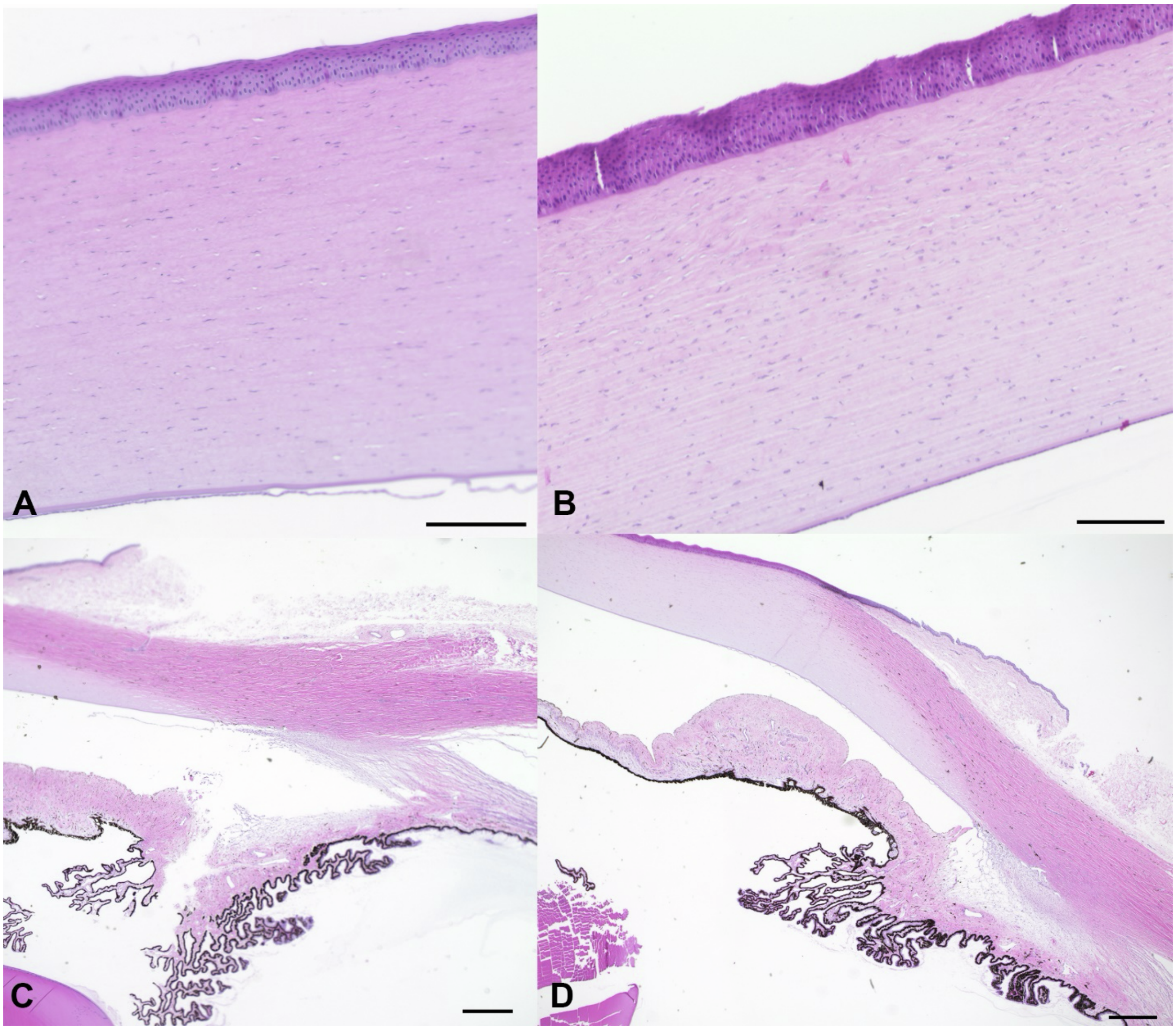

Figure S1: The anterior segment is normal in TSD compared to controls. A,B) Normal corneal morphology in a 9-month-old male TSD sheep (B) compared to a control 9-month male Jacob sheep **(A).** Scale 200 µm. **C,D)** Normal limbus, iris, ciliary body, and ciliary cleft in a 9-month-old male TSD sheep **(D)** compared to a control 9-month male Jacob sheep **(C).** Scale bar 500 µm.

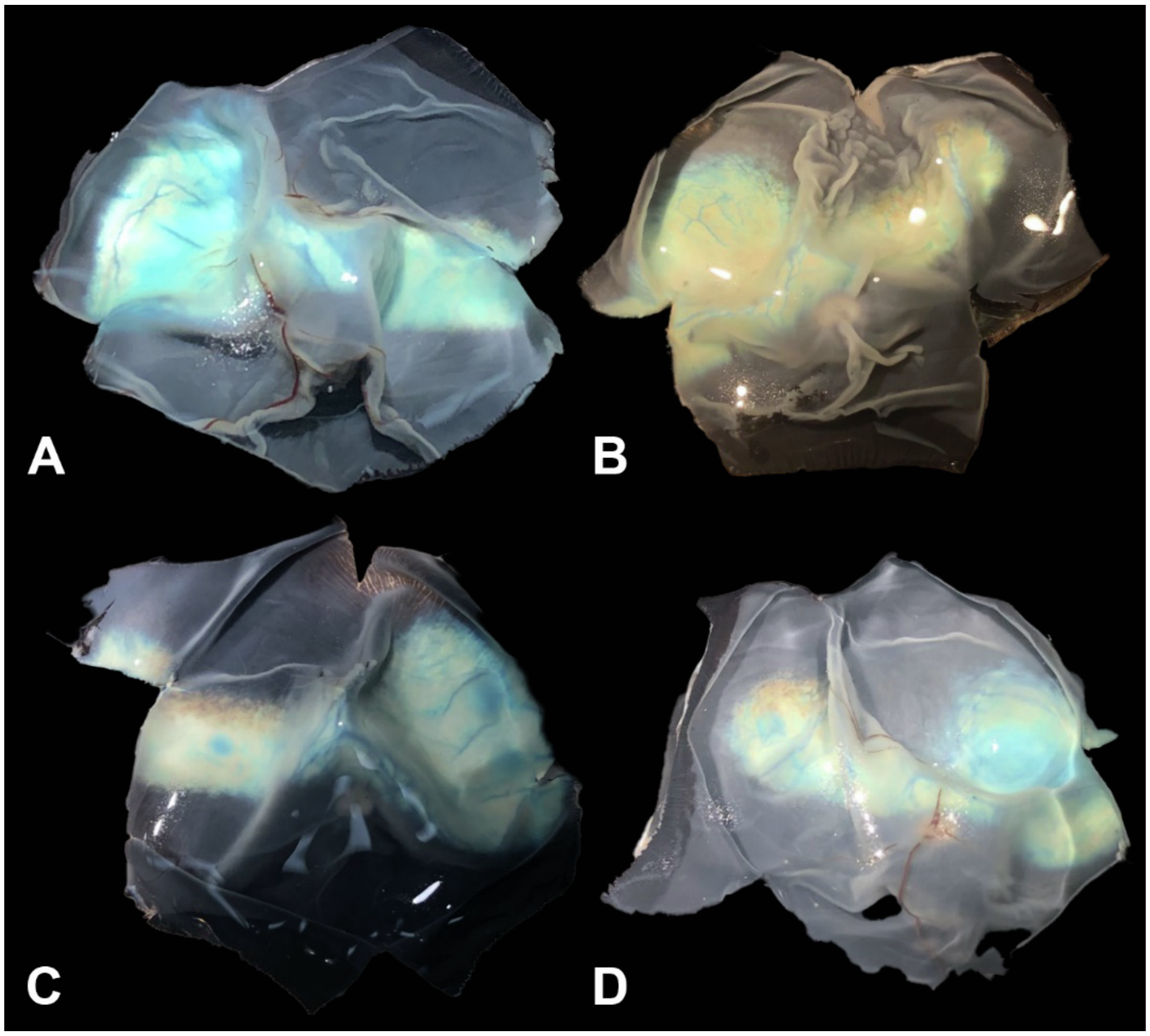

Figure S2: Grossly normal retinal dissections in TSD sheep. A "cherry-red spot" is absent in TSD sheep, given that they lack a macula and fovea. **A&B)** Right and **C&D)** left retinas of TSD-affected sheep.

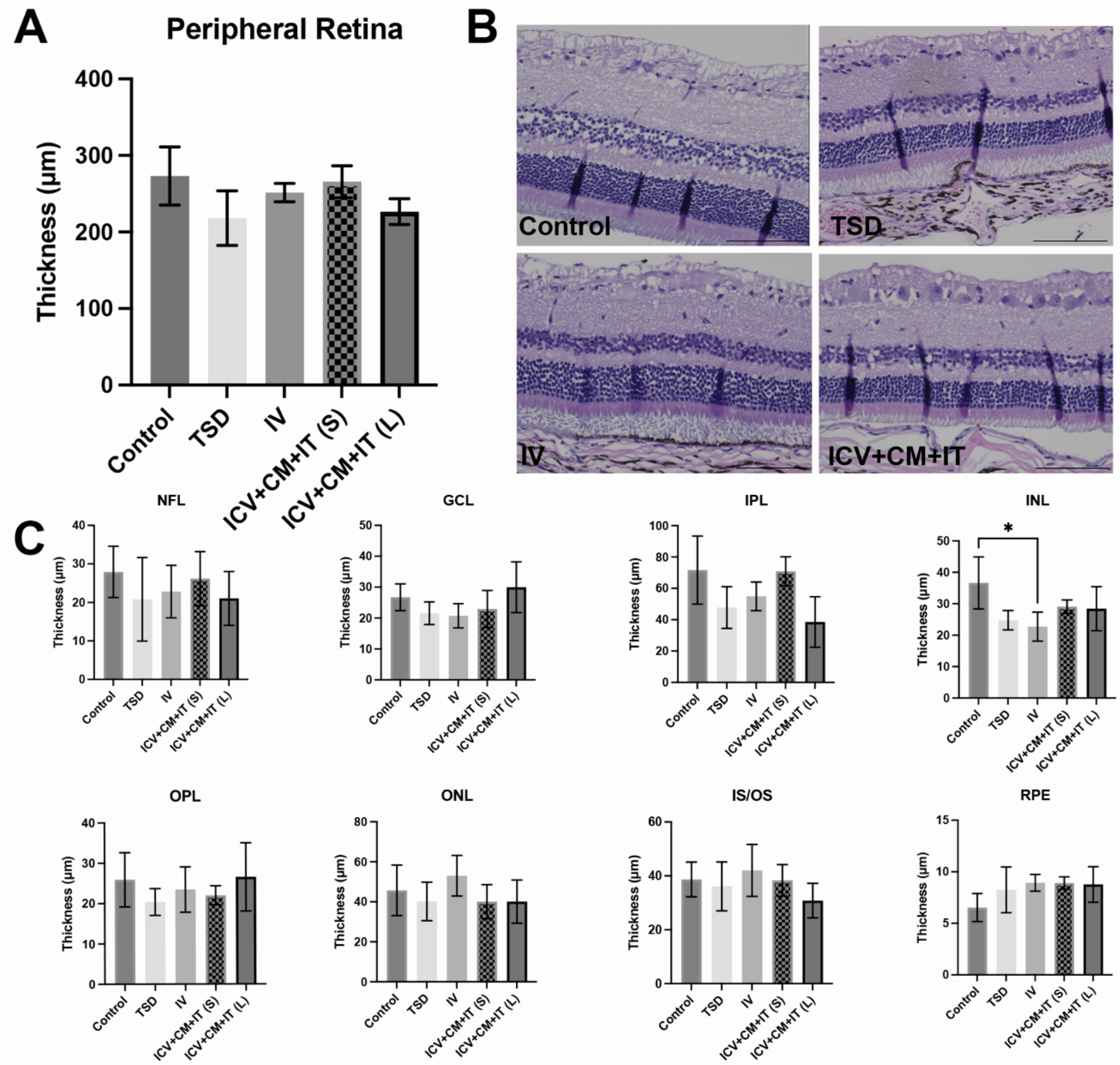

Figure S3: Peripheral retinal thickness measurements showed no appreciable differences except in the INL. A&B) There was no significant difference in total retinal thickness between untreated control sheep and TSD-affected or treated sheep. **C)** When analyzing the individual layers, the only significant difference was observed in the inner nuclear layer. Intravenously treated AAV9 sheep showed a significantly decreased inner nuclear layer thickness compared to controls (*P=0.04*). All other retinal layers showed no significant difference from control sheep (*P>0.05*). Scale bar 100 µm.

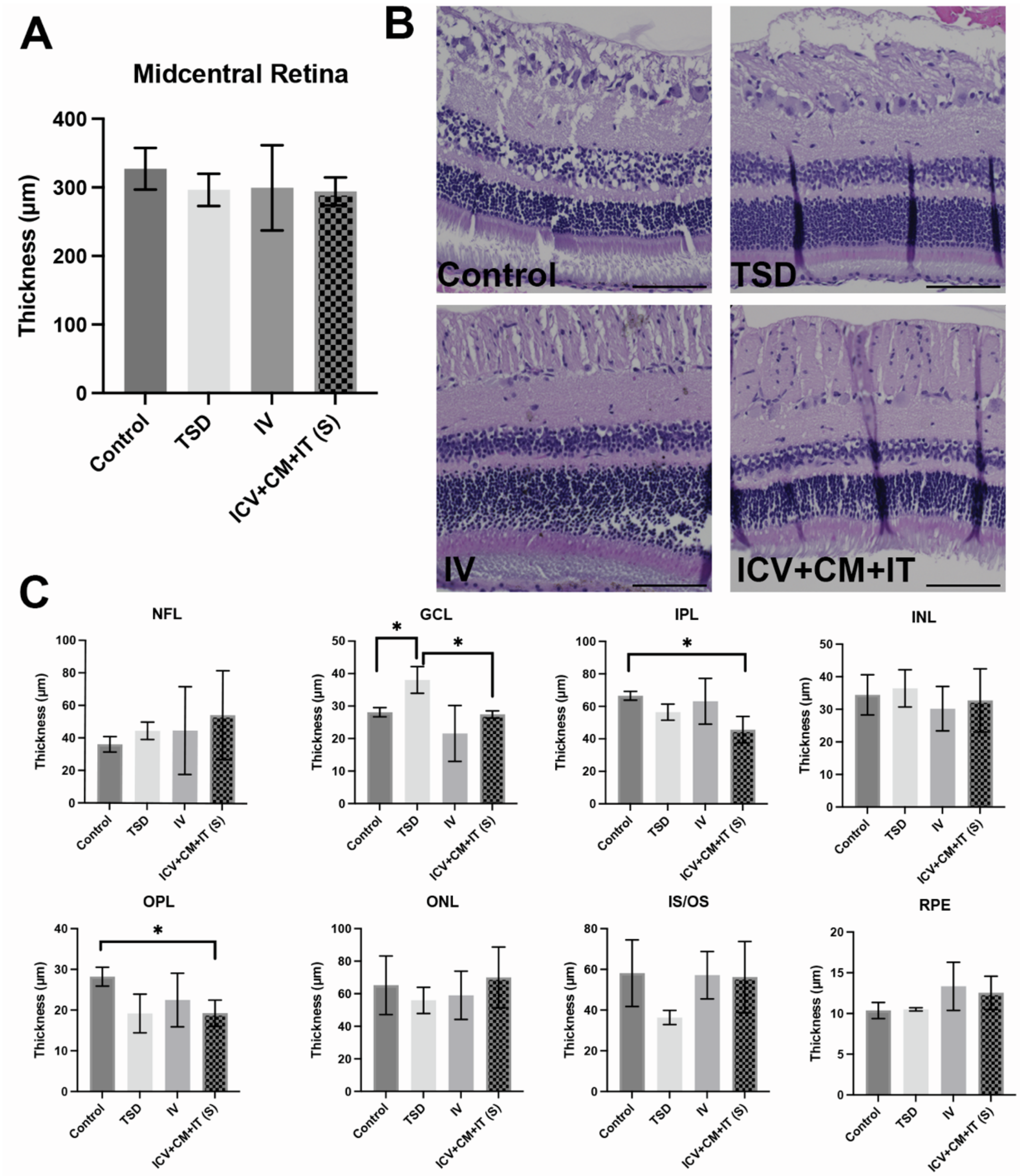

Figure S4: Midcentral thickness measurements showed marginally increased RGCL thickness in TSD sheep. A&B) There was no significant difference in total retinal thickness between untreated control sheep and TSD-affected or treated sheep. C) Small but significant increases in RGCL thickness were seen in TSD-affected compared to controls

and short-term CSF therapy sheep (*P=0.04*). Short-term CSF therapy sheep also exhibit mild but significant decreases in IPL (*P=0.04*) and OPL (*P=0.02*) thickness. All other retinal layers showed no significant difference from control sheep (*P>0.05*). Scale bar 100 μm.

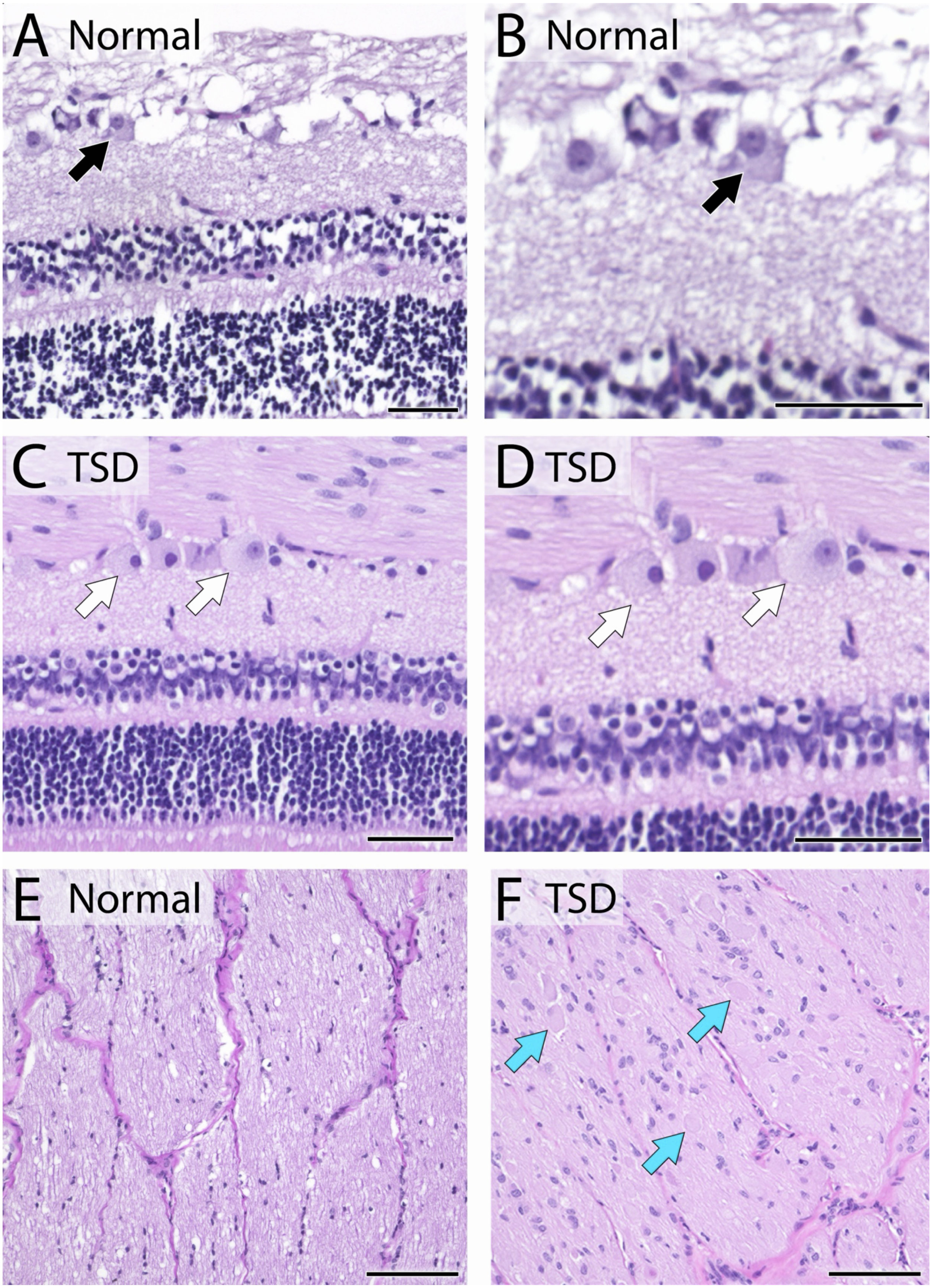
A Normal
B Normal
C TSD
D TSD
E Normal
F TSD

Figure S5: Marked storage material in the RGC cytoplasm with optic nerve spheroids in a sheep affected with Tay-Sachs disease. A, B) Normal ganglion cell layer (black arrows) in a 13-year-old male domestic sheep. **C, D)** Affected retina in a 7.5-month male TSD sheep. Note that retinal layering is well preserved. Storage material distends the cytoplasm of cells in the inner retina, particularly in the ganglion cell layer (white arrows). Outer retinal layers appear unaffected. 20x (A,C) 40x (B,D). Scale 50 µm. **Optic nerve in a normal (E) and Tay-Sachs (F) Jacob sheep. E)** Normal optic nerve parenchyma in a 9-month-old male Jacob sheep. **F)** Multiple neuronal cells exhibit distended cytoplasm with storage material throughout the optic nerve parenchyma (spheroids, purple arrows) in an 8-month female TSD sheep. 20x (E&F). H&E. Scale 100 µm.

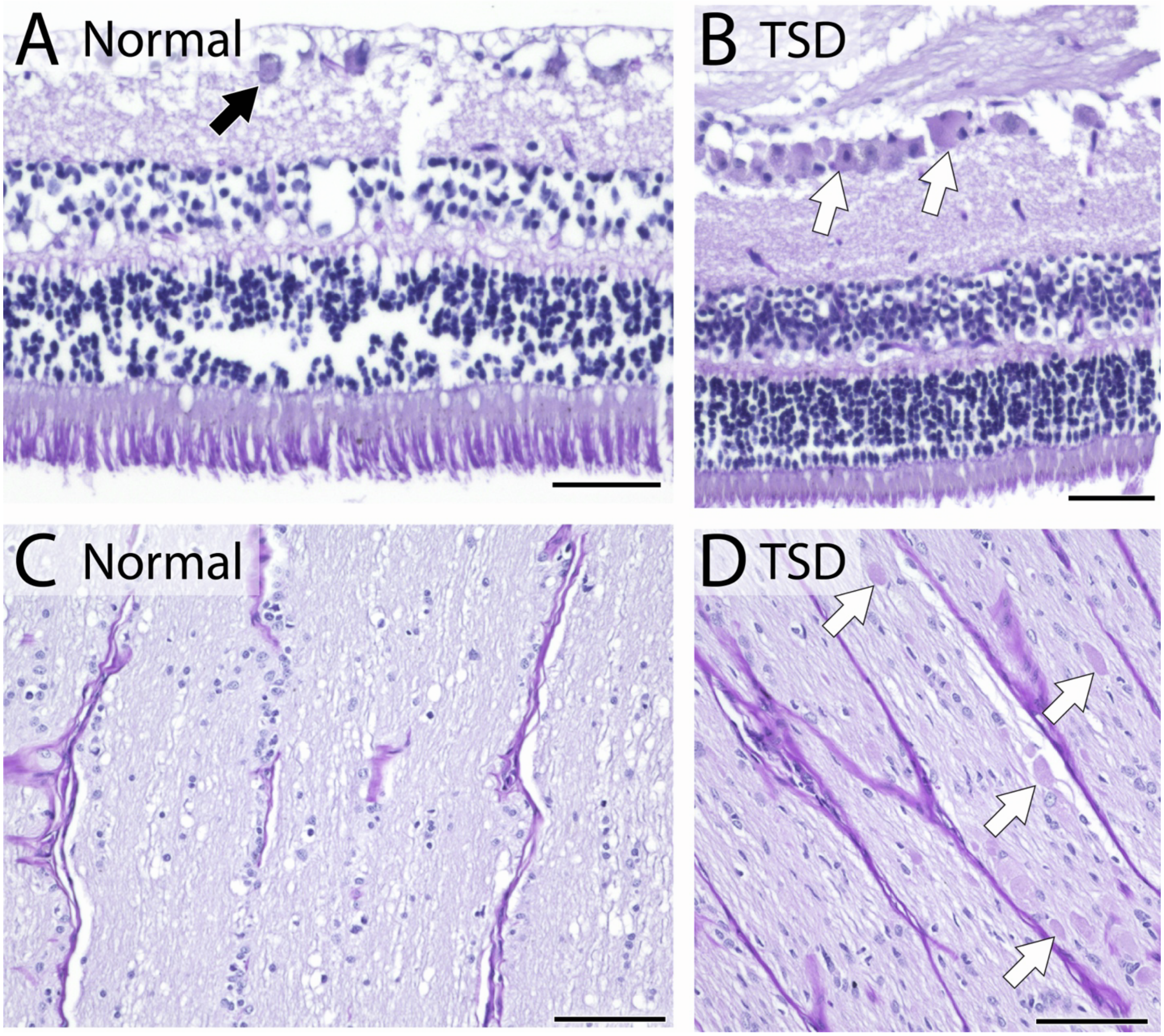

Figure S6: Periodic acid-Schiff labeling of the retina and optic nerve in a normal Jacob sheep and a sheep affected with Tay-Sachs disease showing that the cytoplasm of affected RGCs and cells in the inner nuclear layer stain variably pink with PAS. A) Normal central retinal section of a 13-month-old crossbred male sheep shows minimal RGC cytoplasmic PAS staining. **B)** Central retinal section of a 5-month CSF therapy-treated sheep shows that the variability in PAS stain occurs even between RGCs in the same anatomic region (white arrows). **A)** The RGCs in normal sheep retinas (black arrow) did not stain pink,

or stained very faintly pink, with PAS stain. **C)** No PAS-positive cells could be seen in the optic nerve of normal sheep. **D)** Suspected spheroids (white arrows) of the optic nerve of a 7.5-month TSD untreated male sheep also stain variably pink with PAS. Scale 50 μm (A&B; 20x). Scale 100 μm (C&D; 40x).

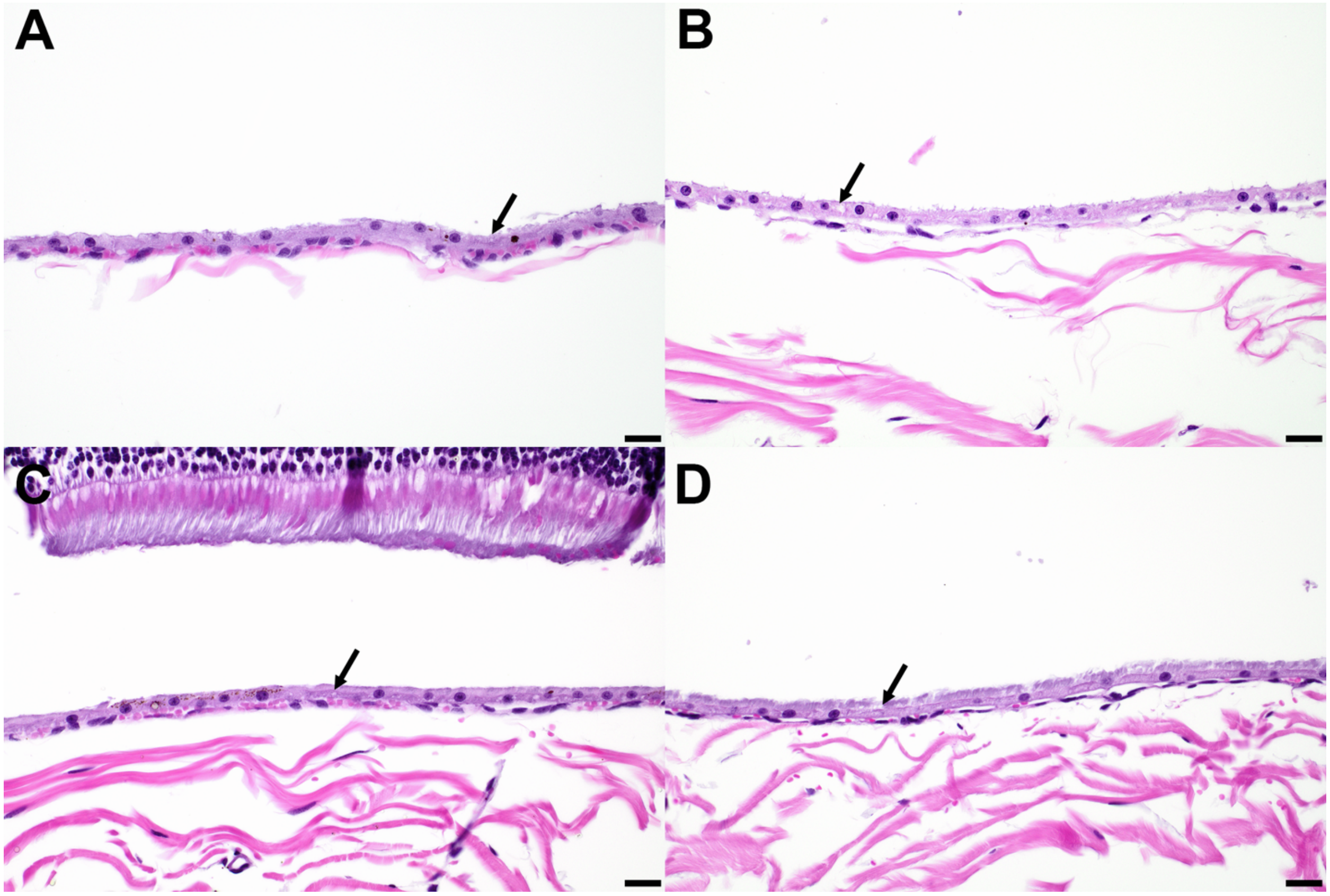

Figure S7: Vacuoles are present in the RPE of control, affected, and treated sheep. RPE vacuolization (black arrows) of unknown significance is present in normal (A), TSD (B), IV (C), and short-term CSF (D) treated sheep. Scale bar 20 μm.

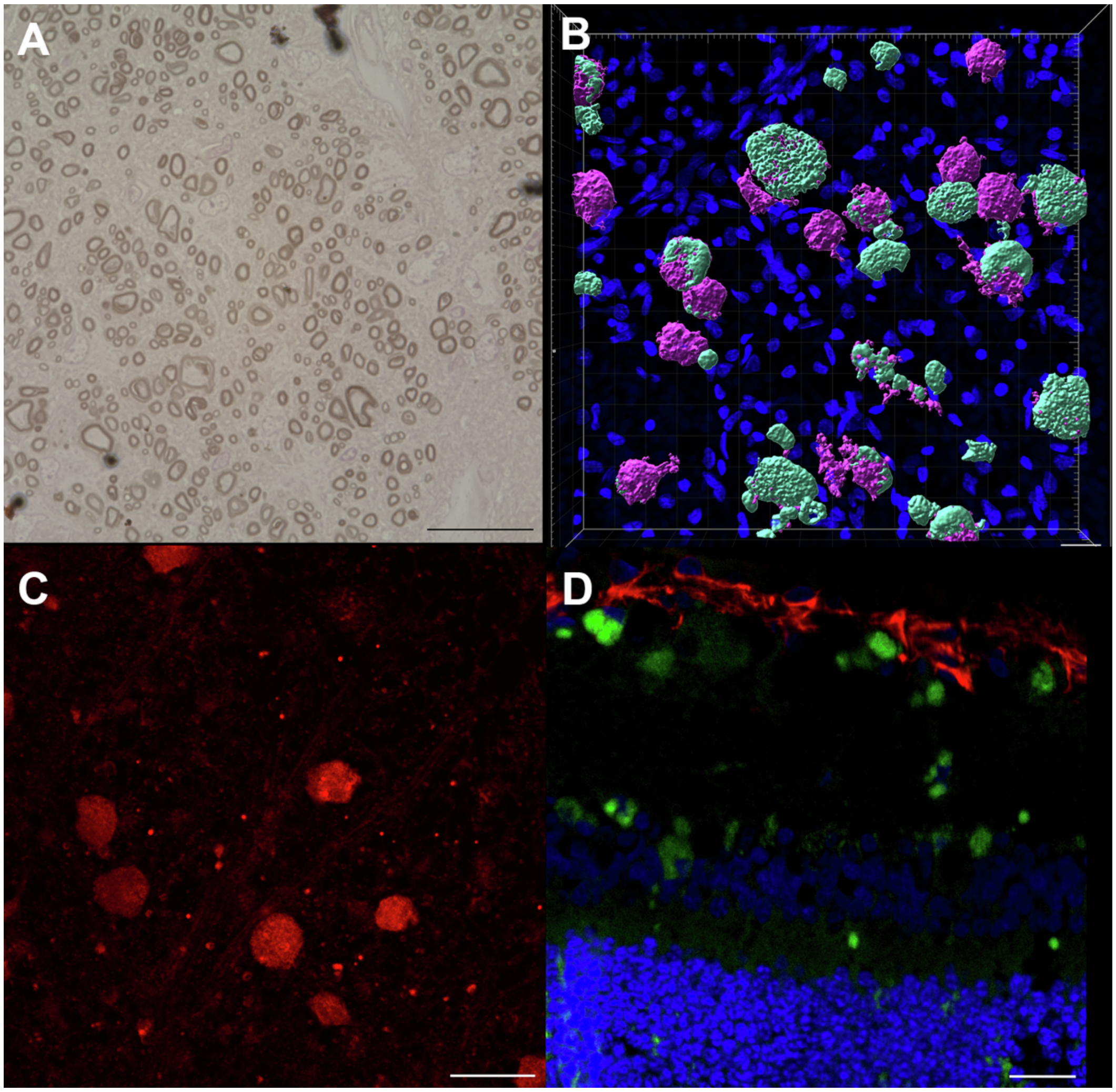

Figure S8: **Representative images from a 47-month-old female long-term CSF-treated sheep illustrate severe pathology. A)** PPD-stained optic nerve showed significant axonal loss (182,527 axons) and increased damage scores (score=4). **B)** Marked GM2 accumulation in RBPMS-positive RGCs. RBPMS = magenta, GM2 = green, DAPI = blue. **C)** Decreased RGC density. RBPMS = red. **D)** Marked inner retinal astrocytosis and microgliosis. Scale bars A&B) 20 µm C&D) 50 µm. GFAP = red, IBA1 = green, DAPI = blue.

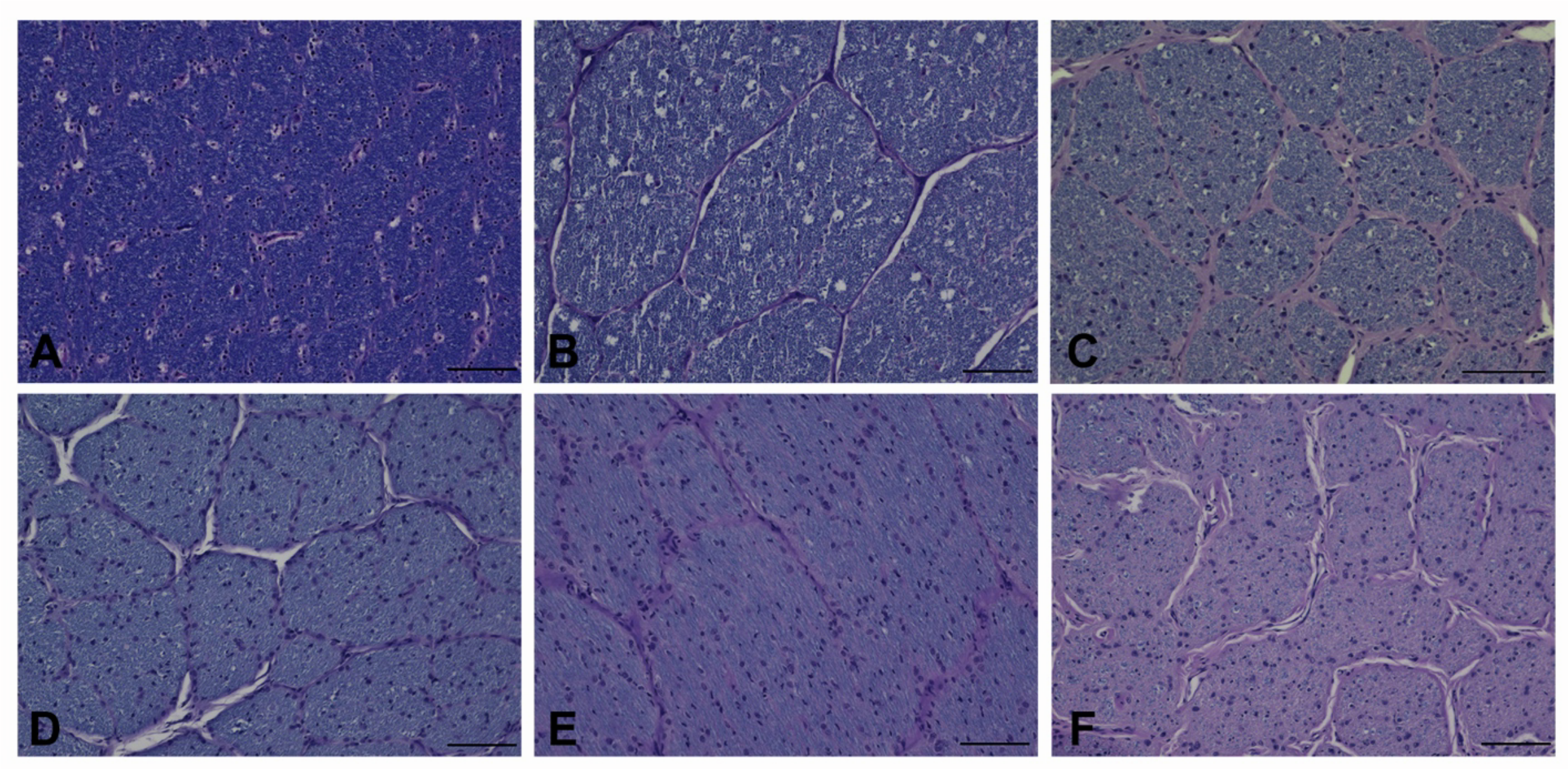

Figure S9: Optic nerves stained with Luxol fast blue show increased myelination in ICV+CM+IT short-term treated sheep. A) Control canine cerebrum. **B)** 7.5-month-old male control sheep. **C)** A 7.5-month-old male untreated TSD sheep shows mildly decreased LFB stain. **D)** A 5-month old male short-term CSF therapy-treated sheep with normal LFB staining. **E & F)** A 44-month-old and a 47-month-old female, respectively, long-term CSF therapy-treated sheep show decreased LFB staining. Scale bar 100 μm.

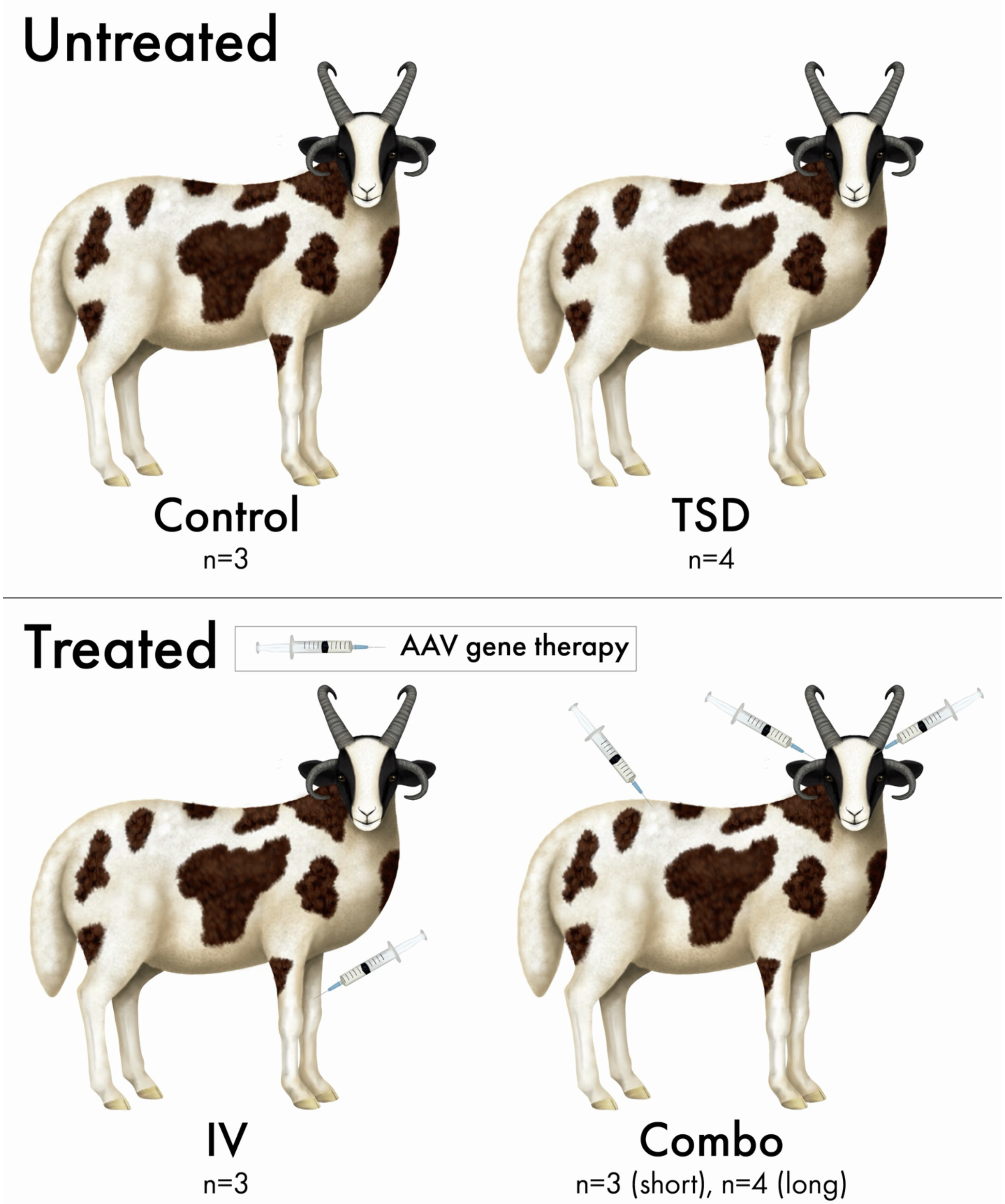

Figure S10: Graphical representation of the sheep cohorts. Control sheep included wer e two 13-month-old crossbred sheep (male and female) and one 6-year-old female Jac ob

sheep. Untreated TSD consisted of a 7.5-month male, an 8-month female, a 9-month female, and a 13-month female. Intravenous therapy sheep (all female) were treated with $5x10^{13}$ vector genomes (vg) per kg at 10, 11, and 13 days of age with humane endpoints of 13.6, 16.3, and 23.7 months. Short-term CSF therapy sheep (two males, one female) were treated with $1x10^{14}$ vg/kg vector genomes (vg) at 23, 24, and 31 days of age with endpoints of 5 months. Long-term CSF sheep (two males, two females) were treated with $1x10^{14}$ vg/kg vector genomes (vg) at 25, 32, 35, and 40 days of age with end points of 10, 45, 47, and 49 months.

Table S1: Hex activity in the retina showed no difference across treatment groups.

Sample	Fluo 1	Fluo 2	Fluo 3	Abs Avg. Corr	nmol 4MU	ssay Time (mi	Assay Time (	nmol/hr	nmol/hr/mL	Protein conc. (mg/mL)	Hex activity (nmol/hr/mg)
174 IV	2236	1898	1525	1728	0.2163282	30	0.5	0.4326565	540.82059	7114.2857	0.07602
194 IV	934	920	914	764.33333	0.0863945	30	0.5	0.172789	215.98621	2280.9524	0.09469
179 IV	961	974	979	813	0.0929563	30	0.5	0.1859127	232.39085	2709.5238	0.08577
184 IV	2030	2028	1955	1846	0.2322385	30	0.5	0.464477	580.59623	9733.3333	0.05965
197 IV		2228	2574	2242.6667	0.2857221	30	0.5	0.5714442	714.3053	6257.1429	0.11416
315 CSF		3604	4071	3679.1667	0.4794093	30	0.5	0.9588185	1198.5231	14828.571	0.08083
215 CSF	2714	2694	2647	2526.6667	0.3240146	30	0.5	0.6480292	810.03649	12019.048	0.06740
318 CSF	2601	2292	2204	2207.3333	0.280958	30	0.5	0.5619161	702.39508	9876.1905	0.07112
271 CSF		1014	1034	865.66667	0.1000575	30	0.5	0.2001151	250.14382	2185.7143	0.11444
259 CSF	1232	1269	1256	1094	0.1308443	30	0.5	0.2616886	327.11081	2828.5714	0.11565
314 CSF	2725	2793	2671	2571.3333	0.3300371	30	0.5	0.6600742	825.09281	7947.619	0.10382
287 CSF	754	813	927	673	0.0740798	30	0.5	0.1481595	185.19942	4138.0952	0.04475
47 WT	1505	1527	1410	1322.3333	0.1616311	30	0.5	0.3232622	404.07779	2923.8095	0.13820
81 WT	4673	4625		4490.6667	0.588826	30	0.5	1.1776519	1472.0649	6828.5714	0.21557
80 WT	2911	2933	2908	2759	0.3553407	30	0.5	0.7106814	888.35181	4257.1429	0.20867
61 WT	2172	2291	2180	2056	0.2605534	30	0.5	0.5211067	651.38338	2304.7619	0.28263
19 TSD	2320	2392		2197.6667	0.2796546	30	0.5	0.5593093	699.13662	5471.4286	0.12778
56 TSD	1637	1511	1481	1384.6667	0.1700357	30	0.5	0.3400714	425.08921	3090.4762	0.13755
22 TSD	2871	2829		2691.6667	0.346262	30	0.5	0.692524	865.65497	4900	0.17666
268 TSD	1628	1605	1554	1437.3333	0.1771369	30	0.5	0.3542737	442.84218	5519.0476	0.08024
57 TSD	1817	1886		1693.1667	0.2116316	30	0.5	0.4232631	529.07891	2185.7143	0.24206

Table S2: The sheep cohorts consisted of normal (n=3), TSD untreated (n=4), IV treated (n=3), and CSF treated (intracerebroventricular, cisterna magna, and lumbar intrathecal administration; n=7 (n=3 short-term, n=4 long-term)). Control sheep included were two 13-month-old crossbred sheep (male and female) and one 6-year-old female Jacob sheep.

Untreated TSD consisted of a 7.5-month male, an 8-month female, a 9-month female, and a 13-month female. Intravenous therapy sheep (all female) were treated with $5x10^{13}$ vector genomes (vg) per kg at 10, 11, and 13 days of age with humane endpoints of 13.6, 16.3, and 23.7 months. Short-term CSF therapy sheep (two males, one female) were treated with $1x10^{14}$ vg/kg vector genomes (vg) at 23, 24, and 31 days of age with endpoints of 5 months. Long-term CSF sheep (two males, two females) were treated with $1x10^{14}$ vg/kg vector genomes (vg) at 25, 32, 35, and 40 days of age with end points of 10, 45, 47, and 49 months.

Sheep	Cohort	Treatment Age (days)	Sex	Dose	Endpoint (months)
N1	Normal	N/A	M	N/A	13
N2	Normal	N/A	F	N/A	13
N3	Normal	N/A	F	N/A	72
197	TSD	N/A	F	N/A	13
268	TSD	N/A	F	N/A	9
393	TSD	N/A	F	N/A	8
394	TSD	N/A	M	N/A	7.5
174	IV	13	M	5E13 vg/kg	21.7
179	IV	11	F	5E13 vg/kg	16.3
184	IV	13	F	5E13 vg/kg	23.7
314	CSF (S)	23	F	1E14 vg	5
315	CSF (S)	24	M	1E14 vg	5
318	CSF (S)	31	M	1E14 vg	5
215	CSF (L)	25	F	1E14 vg	42.2
237	CSF (L)	40	F	1E14 vg	46.6
259	CSF (L)	32	M	1E14 vg	10.1
271	CSF (L)	22	M	1E14 vg	48.5
